# Tendon Tissue Engineering and Its Role on Healing of the Experimentally Induced Large Tendon Defect Model in Rabbits: A Comprehensive *In Vivo* Study

**DOI:** 10.1371/journal.pone.0073016

**Published:** 2013-09-05

**Authors:** Abdolhamid Meimandi-Parizi, Ahmad Oryan, Ali Moshiri

**Affiliations:** 1 Division of Surgery, Department of Clinical Sciences, School of Veterinary Medicine, Shiraz University, Shiraz, Iran; 2 Department of Pathology, School of Veterinary Medicine, Shiraz University, Shiraz, Iran; University of California, Merced, United States of America

## Abstract

Healing of large tendon defects is challenging. We studied the role of collagen implant with or without polydioxanone (PDS) sheath on the healing of a large Achilles tendon defect model, in rabbits. Sixty rabbits were divided into three groups. A 2 cm gap was created in the left Achilles tendon of all rabbits. In the control lesions, no implant was used. The other two groups were reconstructed by collagen and collagen-PDS implants respectively. The animals were clinically examined at weekly intervals and their lesions were observed by ultrasonography. Blood samples were obtained from the animals and were assessed for hematological analysis and determination of serum PDGF level, at 60 days post injury (DPI). The animals were then euthanized and their lesions were assessed for gross and histopathology, scanning electron microscopy, biomechanical testing, dry matter and hydroxyproline content. Another 65 pilot animals were also studied grossly and histopathologically to define the host implant interaction and graft incorporation at serial time points. The treated animals gained significantly better clinical scoring compared to the controls. Treatment with collagen and collagen-PDS implants significantly increased the biomechanical properties of the lesions compared to the control tendons at 60****DPI (*P<0.05*). The tissue engineered implants also reduced peritendinous adhesion, muscle fibrosis and atrophy, and increased ultrasonographical echogenicity and homogenicity, maturation and differentiation of the collagen fibrils and fibers, tissue alignment and volume of the regenerated tissue compared to those of the control lesions (*P<0.05*). The implants were gradually absorbed and substituted by the new tendon. Implantation of the bioimplants had a significant role in initiating tendon healing and the implants were biocompatible, biodegradable and safe for application in tendon reconstructive surgery. The results of the present study may be valuable in clinical practice.

## Introduction

An Achilles tendon defect in the setting of large tissue loss is a more difficult problem than simple repair of a ruptured or lacerated tendon [1]–[3]. Due to many soft connective tissue tumors such as Xantoma, fibrosarcoma and liposarcoma, gangrenous and infective ulcers, burning, traumatic injuries (e.g. car accident or gunshot trauma), tendinitis or tendinopathy, and neglected Achilles tendon ruptures or chronic ruptures [1], [3]–[10], large tendon defect could occur [6]. In such circumstances it is often necessary to resect the remaining Achilles tendon and reconstruct the defect area [6]–[8]. If such a treatment is neglected, then joint stiffness develops and the functionality, especially at ankle area, would be significantly impaired which complicates the condition [4], [6], [8], [9]. However, treatment of large Achilles tendon defect is technically demanding [2], [3], [6]. In fact, variation in surgical techniques for repairing a large Achilles tendon defect is remarkable and it is therefore difficult to determine the method of choice [4], [9], [10]. Several surgical techniques exist, only a few of them have been validated in a strict scientific manner [6]. The v–y technique, local tissue augmentation, turn-down flaps, tendon transfer, free tissue transfer and the use of synthetic materials are some examples. Each has its own significant limitations [2]–[4], [7], [11]. Tendon transplantation could be a potential method of choice; however due to the large size of the harvesting autograft, the donor site morbidity is a major concern and the allografts due to many reasons such as disease transmission (e.g. HIV), rejection and ethical concerns, have not been widely accepted as yet [1], [2], [7]. For these reasons treatment of such massive tendon injuries is a state of art and depends on the surgeon’s experience, equipment, facilitation, and condition [1], [3], [7]. In addition, it should be highlighted that injured tendons have low healing capability and they should tolerate large tensions during the healing process which increase the rate of failure after surgical treatment [5], [12], [13]. Moreover, tendon healing has its own limitations such as development of peritendinous adhesion and muscle fibrosis and in those tendon injuries with significant tissue loss the natural healing response may not be able to replace the damaged tissue [3], [5], [14].

Tissue engineering is an option but because of variable manufacturing technologies, lack of experimental investigations and application of different materials, it is still in its initial stages to be applicable in clinical practice [12], [15], [16]. The *in vitro* investigations have shown promising insights but *in vivo* results are not clear [15].

Basic tissue engineering technologies are limited to acellularization of the tissue grafts [12], [17]–[19] in which, the cellular elements are rinsed from the cadaveric tissue while the architecture of the graft is not altered [10], [13], [19], [20]. The newer technologies used specific molecule(s) such as collagen to produce tissue engineered scaffolds [12], [21], [22]. Such scaffolds can be designed according to the tissue engineering goals in order to be biocompatible, biodegradable and effective in tissue restoration [21], [23]. Most of the tissue engineered scaffolds for tendon healing are manufactured as a film, sheath or membrane in which the scaffold has bidimensional architecture. Such scaffold is wrapped around the injured tendon and its major application is best suited for those tendon ruptures with minimum tissue loss with the aim of controlling peritendinous adhesion [24]. However, for reconstruction of a large Achilles tendon defect, the scaffold should have tridimensional architecture to fill the defect area and mimic the native tissue.

Intact tendons are mainly made up of collagen molecules [5], [25] whereby they are polymerized and arranged as fibrils, fibers, fiber bundles and fascicles to form the highly aligned tridimensional architecture of the tendon proper [12], [13]. Tendon is covered by a paratenon to reduce the extent of attrition in its space during the normal physiological movement [5], [25], [26].

Collagen molecules are the best biomaterial used to reconstruct the injured tissues *in vivo* [12], [13], [15], [16]. These molecules are biocompatible, biodegradable and attract the fibroblasts and inflammatory cells [12], [13], [27], [28]. Collagen can be extracted from xenograft tissues, be architecturally arranged as the target tissue and used in production of desired scaffolds [29], [30]. Synthetic materials such as polydioxanone polymers are used in constructing the bidimensional scaffolds [31]. These materials are biodegradable, biocompatible and are absorbed differently compared to biologic molecules such as collagens [31]–[33]. They are absorbed more slowly than collagen; therefore they may be applicable as tendon sheaths because they can act as a barrier for peritendinous fibroblasts and probably reduce the extent of peritendinous adhesion during the healing processes of the injured tendons [26], [31].

We used collagen molecules and artificially produced an aligned tridimensional scaffold to mimic a tendon proper. To simulate a paratenon, a polydioxanone bidimensional scaffold was wrapped around the collagen scaffold. Therefore, this study was designed to test whether the collagen based tridimensional bioimplant, with or without polydioxanone scaffold, is/are effective in producing a new tendon in an experimentally induced large Achilles tendon defect model in rabbits.

This experiment was designed based on the following hypotheses: 1) Are the scaffolds biocompatible and biodegradable? 2) Is the collagen scaffold acutely rejected or incorporates in the healing tissue? 3) What is the role of alignment of the collagen implant in promoting tendon healing? 4) Does the collagen implant and PDS sheath motivate the inflammatory response? 5) What is the role of this inflammation in tendon healing and tissue restoration? 6) Do the implants reduce peritendinous adhesion during tendon healing? 7) Is the collagen scaffold replaced by a new tendinous tissue? 8) What are the roles of these implants on collagen content and biomechanical properties of the newly regenerated tissue? 9) How do the implants manage the extrinsic and intrinsic mechanisms of tendon healing and which mechanism is more involved in the healing of such large tendon defects? 10) Is the treatment strategy able to improve the function of the injured limb?

## Materials and Methods

### Ethics

The investigators who undertook the measurements and analyses of the results were unaware of the experimental design and grouping details. All operative procedures were performed by one surgeon. All animals received humane care in compliance with the Guide for Care and use of Laboratory Animals published by the National Institutes of Health (NIH publication No. 85-23). The study was approved by Ethic Committee of “Regulations for using animals in scientific procedures” in Veterinary Medicine School of Shiraz University, Shiraz, Iran.

### Study Design

Simple, interventional, double control, randomized, experimental, *in vivo*, study.

### Preparation of the Collagen Implant and Polydioxanone Sheath

**Figure 1:**
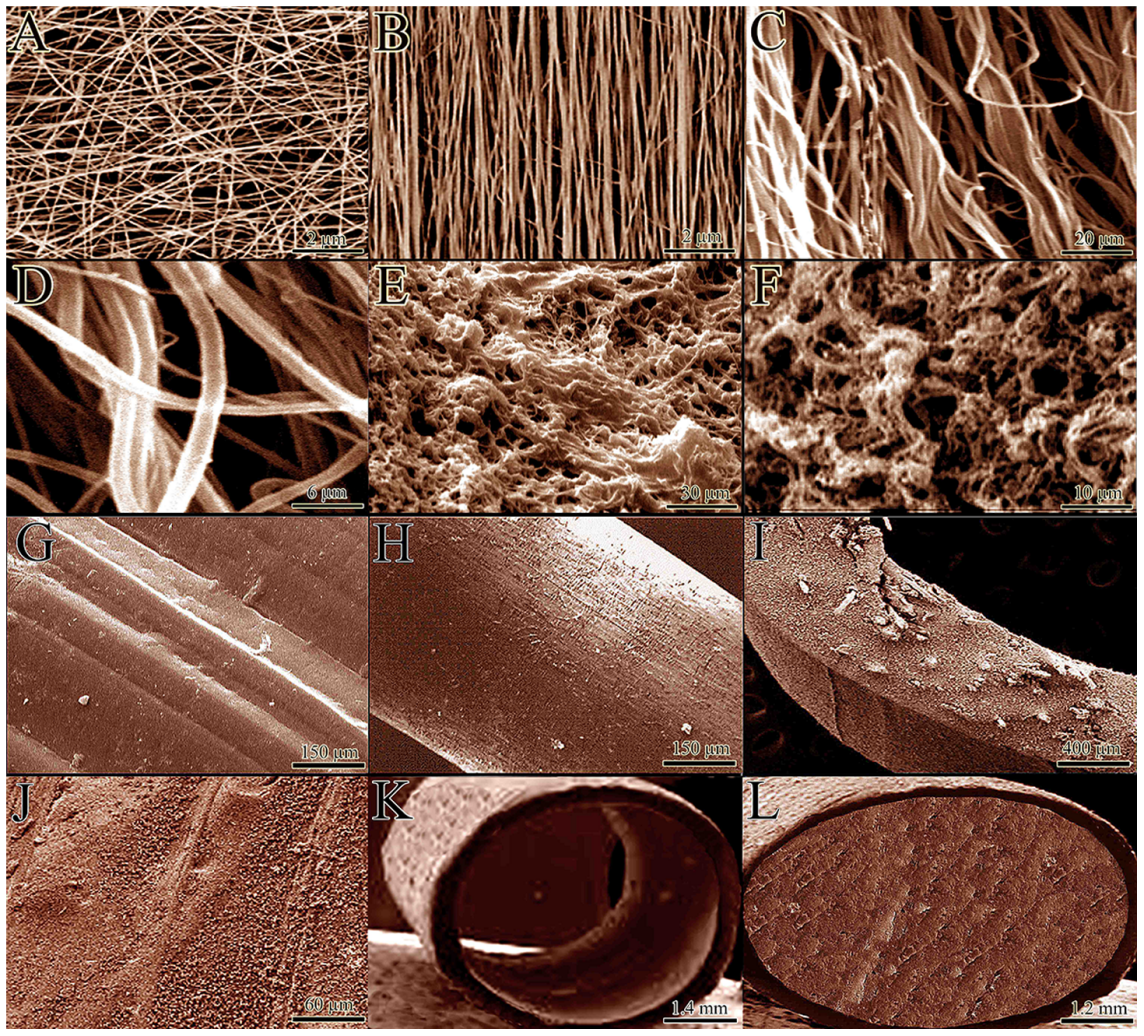
Morphology of the implants. A: Non-aligned electrospun collagen fibers constructed by electrospinning without dual plate device. B: Aligned electrospun collagen fibers were produced, using gap collector. C: Aligned internal architecture of the polymerized collagen fibers in the gel. D: The composition of the collagen implant at a larger magnification. E and F: Transverse section of the collagen implant after crosslinking. Most of the implant is filled with the polymerized collagen fibers while little porosity exists between these fibers. G: Surface of the collagen implant after crosslinking. H–J: Surface of the polydioxanone sheath. Note that there is no porosity in the external and internal architecture of the polydioxanone sheath. K: Circular polydioxanone sheath. L: Polydioxanone plate was melted and wrapped around the collagen implant to produce a collagen-PDS scaffold. PDS sheath could be seen at periphery while the collagen implant is seen in the center.

Collagen type I was extracted from the bovine superficial digital flexor tendon according to the methods of Foltran et al [29]. The purity of the type I collagen was confirmed by SDS/PAGE [29]. The acid solubilized collagen molecules were electrospinned onto a dual plate device to produce the large and aligned electrospun collagen fibers according to the methods of Wray et al [34] (Fig. 1 A and B). After electrospinning, the acid-solubilized bovine tendon type I collagen molecules were mixed with electrospun collagen fibers and polymerized in an incubator at 4°C for 48 hours to produce tridimensional collagen gel. The collagen fibers were aligned under 12 Tesla magnetic fields (CRETA, Grenoble) during polymerization according to the methods of Dubey et al [35]. The electrospun collagen matrix (2 D) acted as a core with its fibro-conductive characteristics and improved the alignment of the newly formed collagen fibers (Fig. 1 C–F). The collagen composite was cut into several pieces of the same size and shape as the rabbit’s Achilles apparatus (L = 2 cm, H = 3.5 mm, W = 3 mm). The collagen composites were cross-linked after suspension in iso-osmolar 0.1% riboflavin solution, using UV (wavelength of 365 nm) irradiation according to the methods of McCall et al [36] (Fig. S1 and S2). To produce the bio-synthetic implant, the nano thickness polydioxanone plates (300 nm thickness) were purchased (PDS plate, Ethicon, Johnson & Johnson, USA), melted and wrapped around each collagen piece to cover the peripheral areas of the implant (Fig. 1 H–L). The final product was repeatedly washed with distilled water and received 100 Gray g-radiation and suspended in ethanol 96% to be sterilized and maintain its sterility until surgery [23]. The morphology of the scaffold was studied by SEM. The sterility status and endotoxin content of the implants were tested and confirmed by microbiological and LAL tests *in vitro*, respectively [37]. The scaffolds were seeded by rat skin fibroblasts and the cell viability was determined and confirmed by histology, SEM and live/dead cell assay according to the methods of Tsai et al. [21]. For more information, see Protocol S1 and Fig. S3.

### Animals and Grouping Details

Sixty skeletally-mature male White New Zealand rabbits of 12±2 month’s age and 3.24±0.16 kg body weight were randomly selected for this experiment. The left hind leg of each rabbit was selected to make a tendon defect and the right one was left intact. The animals were randomly divided into three groups of control (*n = 20*), treated with collagen implant (*n = 20*) and treated with collagen-polydioxanone implant (*n = 20*). The left tendon of the control group was named “Injured Control Tendon (ICT)”. The left tendon of the treated group with collagen implant was named “Injured Treated Tendon with Collagen implant (ITTC)” and that of the treated group with collagen-PDS implant was named “Injured Treated Tendon with Collagen- Polydioxanone implant (ITTC-PDS)”.

Another 65 rabbits were used as a pilot model and divided into three groups of control (n = 15), treated with collagen implant (n = 35) and treated with collagen PDS implant (n = 15). Each of the control pilot and the collagen-PDS pilot groups were then divided into three groups of 10, 20 and 30 days post injury (DPI) observation. The treated with collagen implant pilot-group was divided into 7 equal groups of 5, 10, 15, 20, 25, 30, and 40 DPI observation. At the mentioned time points the pilot animals were euthanized and their tendons assessed for gross morphology and histopathology with the aim of following the host-implant interaction of the collagen implant and PDS sheath. In addition, the peripheral blood profile of the pilot animals (*n = 5* in each group) was determined, at 10 DPI.

Each rabbit was housed in individual standard rabbit cage and maintained on standard rabbit diet, with no limitation of access to food or water.

### Premedication and Anesthesia

Premedication was provided by intra-muscular injection of 1 mg/kg Acepromazine maleate and the animals were anesthetized by intra-muscular injection of 15 mg/kg Ketamine combined with 0.05 mg/kg Xylazine hydrochloride (all from, Alfasan Co., Woerden, The Netherlands) [14], [38], [39].

### Injury Induction and Surgical Reconstruction

**Figure 2:**
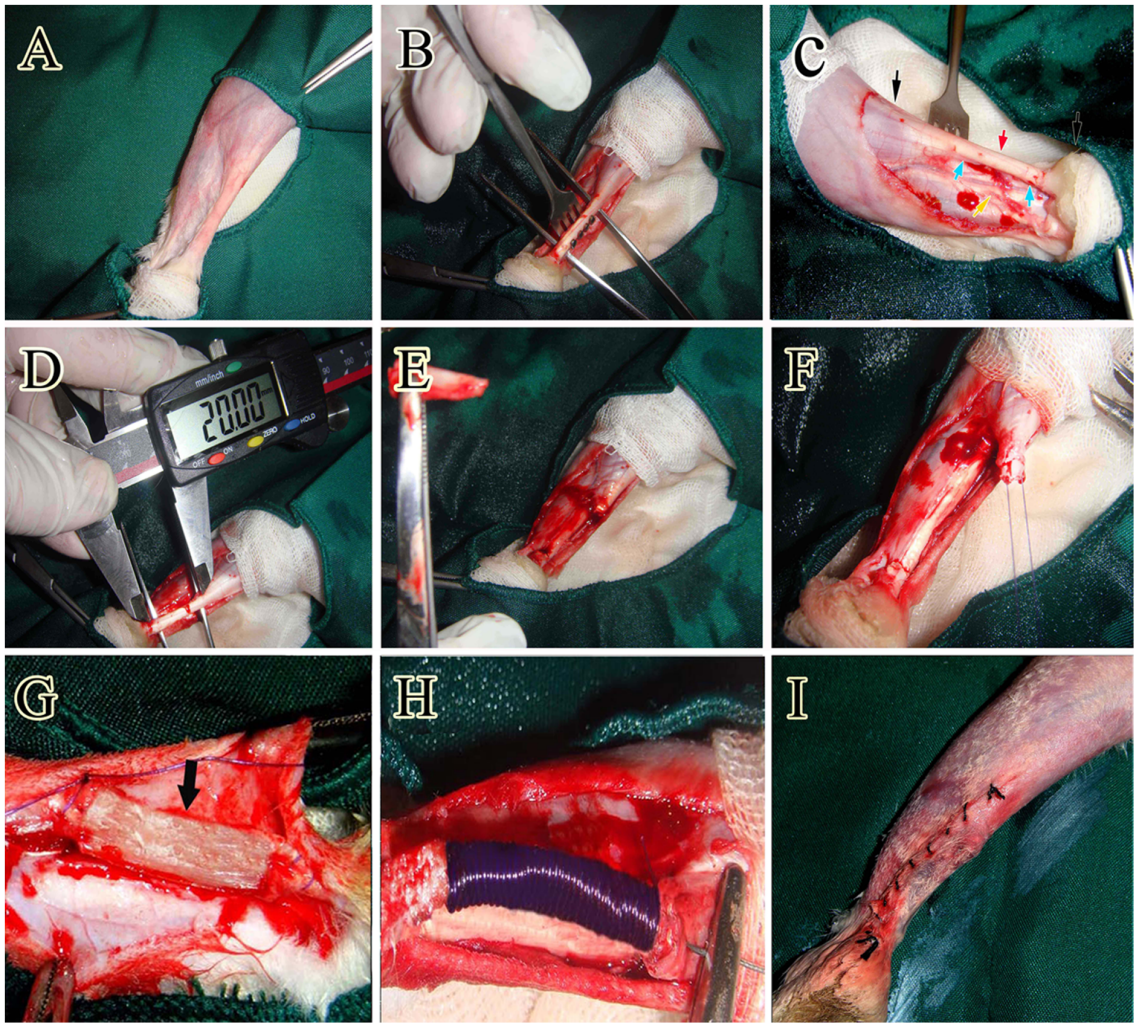
Surgical intervention and implantation of the bioimplant. A: The surgical site and preparation method. B: Skin incision and exposure of the Achilles apparatus. C: Black arrows show the gastro soleus muscle proximally and calcaneal tuberosity distally. The red arrow shows the Achilles apparatus, blue arrows the segment to be removed and the yellow arrow the *tibialis posterior* tendon. D and E: 2 cm of the Achilles tendon was measured with a digital caliper and removed. F: A modified Kessler Core pattern suture was anchored in the ends of the remaining tendon. G and H: The collagen and collagen PDS implants were inserted. I: The skin over the lesion was closed.

Under aseptic condition, a longitudinal skin incision was made over the Achilles apparatus (plantar surface). Then, 2 cm of the Achilles tendon (approximately 75% of the total Achilles apparatus) together with the covering paratenon were excised by transverse incisions, approximately 5 mm distal to the gastrocnemius muscle and 5 mm proximal to the calcaneal tuberosity. Primary reconstruction of the tendon extremities was conducted using double stranded modified Kessler core pattern, by monofilament absorbable Polydioxanone suture material (PDS 0–4, Ethicon, INC.1997, Johnson & Johnson, USA). This aligned the remaining Achilles tendon extremities in a normal anatomical position and created a 2 cm gap between the extremities [14]. The same method was applied to all groups. In the control lesions, the gap was left intact (no implant was applied). In the treated lesions, the tridimensional collagen implant and the tridimensional collagen implant+bidimensional PDS scaffolds were inserted in the defects, respectively. For insertion of the prosthetic implants into the gaps, the double stranded suture was passed through the longitudinal axis of the implant. The skin over the lesion was closed routinely. Postoperative analgesia with fentanyl (Matrifen, Roskilde, DK; 0.0015 mg/kg/h) was provided for 3 days via a transdermal patch applied to the depilated and sutured skin. Due to strict aseptic surgery and the sterility of the implants, no prophylactic antibiotic was used and no wounds became infected (Fig. 2).

### Clinical Examinations

Physical and behavioral status of the animals were weekly monitored (*n = 20* in each group). Tarsal flexion degree (both in cage and on ground), weight distribution per limb (both in cage and on ground), pain on palpation, heel and toe position (both in cage and on ground) and swelling of the injured area were weekly monitored and scored according to the methods of Oryan et al. [40]. The criteria for scoring are shown in Table S1. The sums of the weekly scores were statistically analyzed to define the differences between the groups.

Based on the methods of Oryan et al. [41] the transverse diameter of the injured area (*n = 20* in each group) including healing tissue, subcutaneous and overlaying skin, were measured, using a micrometer measurement device (Guanglu electronic digital caliper, Anyang, South Korea), before injury, and then weekly until end of the experiment. The surface temperature of the skin over the lesion and the contralateral part (*n = 20* in each group) was measured by a laser heat-detector device (Mastech, MS6530 Infrared Thermometer, Seoul, South Korea), at weekly intervals [39].

### Ultrasonography

The injured and normal contralateral tendons (*n = 20* in each group) of all animals were evaluated by ultrasonography at weekly intervals. Transverse and longitudinal (the probe was placed parallel with the Achilles tendon) ultrasonographic images of the tendons were obtained with a 7.5–12 MHz linear probe (Simense SLR-400, Berlin, Germany; Echo wave 3.23 software). Echogenicity, homogeneity, peritendinous adhesion, the quality of the healing and volume of the regenerated tissues were assessed and scored. The criteria for scoring are shown in Table S2 [42].

### Hematology

Immediately before euthanasia, 10 ml blood sample was collected from each animal (*n = 20* in each group, n = 5 in each pilot group) and 1 ml/animal was transferred into EDTA tube and indirectly tested for different cell typing, using standard clinical cell-counter (Veterinary Auto-analyzer, Cambridge, UK). The remaining 9 ml was placed in a regular tube (without EDTA) and centrifuged at 1440 g¸ for 10 minutes to collect the serum. The serum PDGF concentration was measured, using commercially available ELISA kit: Platelet-Derived Growth Factor AA (PDGF-AA) and AB Immunoassay (Biotrend Chemicals, LLC 136, South Holiday Road, Destin, FL 32550) according to the methods of Czarkowska-paczek et al [43].

### Euthanasia

The animals were anesthetized by intra-cardiac injection of a combination of 15 mg/kg Ketamine, 2 mg/kg Xylasin, and 1 mg/kg Acepromazine maleate (all from, Alfasan Co., Woerden, The Netherlands). The anesthetized animals were euthanized by intra-cardiac injection of 1 mg/kg Gallamine triethiodide (Specia Co., France). This last drug depresses the pulmonary muscles and stops breathing [44], [45].

### Sample Collection

Each injured tendon and its contralateral intact tendon was dissected and assessed for gross pathology (*n = 20* left and *n = 20* right in each group). The samples from each group were randomly divided into two equal subgroups. Subgroup 1 (*n = 10* left, *n = 10* right) was used for determination of biomechanical properties. Subgroup 2 (*n = 10* left, *n = 10* right) was divided longitudinally into three parts. Part A (medial part) was used for ultrastructural analysis (SEM), part B (middle part) for histology and part C (lateral part) for assessment of the dry matter and hydroxyproline contents [44], [45].

### Gross Pathology

Each injured tendon (*n = 20* in each group, n = 5 in each pilot group) and NCT (*n = 20* in each group, n = 5 in each pilot group) was evaluated for color changes, adhesion to the surrounding tissues and any other abnormalities. Each tendon was photographed and the images were transferred to computer software (Adobe Photoshop CS-5 Extended, CA, USA) and analyzed by computerized morphometry. Hyperemia, peritendinous adhesion, general appearance, muscle atrophy and fibrosis together with the tendon diameter were then measured and scored using the methods of Moshiri and Oryan [14] and Oryan and Moshiri [42]. The criteria for scoring are shown in Table S3.

### Histopathologic and Histomorphometric Analysis

After routine preparation of the samples (n = 10 left, n = 10 right in each group) according to the methods of Oryan et al [39] they were stained by hematoxylin and eosin and examined by a light microscope (Olympus, Tokyo, Japan). The photomicrographs were recorded by a digital camera (Sony T-700, Tokyo, Japan) and transferred to the computer software (Adobe Photoshop CS-5, CA, USA) for digital analysis. Totally 250 photomicrographs were provided from the injured area of each group (10 tendon×5 tissue section×5 microscopic field) [40].

The cellularity, immature tenoblasts, mature tenoblasts, tenocytes, neutrophils, lymphocytes, macrophages, plasma cells and blood vessels of the tendon proper were counted at ×200 magnification. The transverse diameter of the immature and mature tenoblasts, tenocytes, small, medium and large blood vessels was measured by computer software (Adobe Photoshop CS-5, CA, USA). The collagen mass density was analyzed by computer software (Image J, NIH, CA, USA) and reported as percentage. Crimp pattern, alignment, tissue maturity and perivascular edema were scored [44]. The criteria for scoring are shown in Table S4.

### Scanning Electron Microscopy

The samples were fixed in 2.5% glutaraldehyde, sectioned longitudinally and transversely and then coated by hexa-methyl-di-silazane (TAAB, Co., London) to improve the quality of SEM images. The samples were vacuumed and gold-coated on a Cu mount and were then examined by a scanning electron microscope (Cambridge, London, UK). Different magnifications from ×50 to ×30,000 were used to analyze the morphological and morphometrical characteristics of the implants and tissue samples (*n = 10* for left, *n = 10* for right in each group). Number of cellular elements (at ×10,000), diameter of collagen fibrils (at ×30,000) and collagen fibers (at ×5,000) were then measured. Alignment, crimp pattern, collagen differentiation and density of the collagen fibrils and fibers were scored and analyzed. The criteria for scoring are shown in Table S5.

### Dry Matter Content

The samples obtained from the ICTs, ITTCs and ITTC-PDSs and their NCTs were weighed immediately after euthanasia, and freeze-dried (Helosicc, Ink, Co. London) until a constant weight was obtained and the percentage dry weight was then calculated according to the following equation: dry weight/wet weight × 100 [14], [38].

### Hydroxyproline

The hydroxyproline concentration was measured by spectrophotometry [46]. Samples from mid splitted area of the injured and normal tendons of each animal from the treated and control groups were collected and their hydroxyproline concentration was measured. The samples were hydrolyzed in 6 M HCl at 105°C for 14 h and the hydroxyproline was oxidized by chloramines T, and then by adding Ehrlich’s reagent and incubating at 60°C, a chromophore was formed. To remove the interfering chromophore, the hydroxyproline product in alkaline media was extracted into toluene and then into acid phase. The absorbance of acid phase was read at 543 nm and the hydroxyproline content was calculated from the calibration curve based on the standard solutions run the same as the samples.

### Biomechanical Testing

Biomechanical testing was performed according to the previous methods [39], [42]. Briefly; each tendon was mounted between the two cryoclamps. Biomechanical test was performed using a tensile testing machine (Instron® Tensile Testing Machine, London). Each tendon was loaded by elongating it at a displacement rate of 10 mm·s^−1^ until a 50% decrease in load was detected. Load and crosshead displacement data were recorded at 1500 Hz, and a load-deformation curve was generated for each specimen using Test-Works 4 software (SUME Systems Corporation). The ultimate load, yield load, stiffness, maximum strain, yield strain, maximum stress and modulus of elasticity were determined.

### Statistical Analysis

After application of the normal distribution test, significant differences of the measured values within groups were statistically tested using paired-sample *t-*Test. Significant differences between the groups were tested using One Way ANOVA. Kruskal-Wallis *H* Test was performed to analyze the base-scoring system described in the methods (Table S1–S5). Statistics were performed using the computer software SPSS version 19 for windows (SPSS Inc., Chicago, USA). Differences of *P<0.05* were considered significant. The quantitative data were expressed as: mean and standard deviation (SD). The qualitative scoring data were expressed as: median (minimum-maximum).

## Results

### Clinical Findings

The treated animals with Collagen-PDS prosthesis and those treated with collagen implant significantly gained better scoring number for tarsal flexion degree of the injured limb (18 (16–20) ^Collagen-PDS^ *vs*. 20 (18–24) ^Collagen^ *vs*. 27.5 (23–31) ^Control^, *P = 0.001*, *P = 0.049*), heel and toe position of the injured limb (12 (9–14)^ Collagen-PDS^ *vs*. 14 (12–18)^ Collagen^ *vs*. 19 (17–22) ^Control^, *P = 0.001, P = 0.049*), weight distribution on the legs (20 (16–25)^ Collagen-PDS^ *vs*. 22.5 (18–25)^ Collagen^ *vs*. 29 (26–32)^ Control^, *P = 0.001*, *P = 0.042*) and pain on palpation (12 (11–15)^ Collagen-PDS^ *vs*. 14 (12–17)^ Collagen^ *vs*. 20 (18–24) ^Control^, *P = 0.001*, *P = 0.047*) compared to those of the control animals.

**Figure 3:**
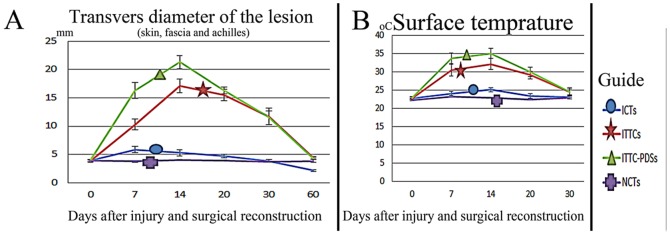
Clinical investigation. A: transverse diameter of the lesion (skin, fascia and Achilles apparatus): The diameter of the ICTs significantly increased (*P<0.05*) at 7 DPI and was elevated at 14 DPI, compared to the NCTs. At 14 to 30 DPI, the diameter of the injured area decreased continually so that it reached its normal value at 30 DPI. From day 30 to 60 DPI, the diameter of the ICTs significantly decreased and was lower than normal value. The diameter of the ITTCs and ITTC-PDSs significantly increased during the first 14 DPI with the peak level at 14 DPI. The measured value of these tendons was significantly higher than the ICTs at 14 DPI. Also, the measured value of the ITTC-PDSs was significantly higher than those of ITTCs at the same stage. From 14 to 60 DPI, the diameter of the injured area of the ITTCs and ITTC-PDSs continually decreased and reached their normal level at 60 DPI. B: Surface temperature of the injured area from the skin over the lesion. The temperature of the ICTs significantly increased during the first 14 days after injury compared to intact tendons. Then after, it decreased to the normal level that was observed in intact tendons, at 20 DPI. The temperature of the ITTCs and ITTC-PDSs significantly increased during the first 14 DPI but the measured value for ITTC-PDSs was significantly higher than those of ITTCs at this stage. The temperature of the ITTCs and ITTC-PDSs, although significantly higher than ICTs at 20 DPI, showed no significant differences between ITTCs and ITTC-PDSs at 20 DPI. At 30 DPI, the measured value for these two last tendons was not statistically different compared to the intact tendons.

At 7 DPI the transverse diameter of the injured area (skin+subcutaneous fascia+Achilles apparatus) significantly increased, compared to 0 DPI (*P = 0.001* for all), in all groups. The transverse diameter of the ITTC-PDSs was also signficantly higher than the ITTCs and both of them were significantly higher than the ICTs, at 7 DPI (*P = 0.001* for all). Unlike ICTs, compared to 7 DPI, the transverse diameter of the ITTCs and ITTC-PDSs significantly increased (*P = 0.001* for both) at 14 DPI but the ITTC-PDSs had significantly higher transverse diameter than the ITTCs at this stage (*P = 0.001*). The transverse diameter of the ICTs signficanly reduced and reached its normal level at 30 DPI compared to those of 7 and 14 DPI (*P = 0.001*, *P = 0.023*). The diameter of the ITTCs and ITTC-PDSs also signficantly reduced compared to the 20 DPI (*P = 0.001* for both). At this stage the diameter of the ITTCs and ITTC-PDSs were signficantly higher than the ICTs and NCTs (*P = 0.001* for all). At 60 DPI, the diameter of the ITTCs and ITTC-PDSs reached their normal level and there were no significant diffrences between these tendons at 60 DPI compared to those of the 0 DPI and their NCTs (*P>0.05* for all). At this stage, the transverse diameter of the ICTs was signifcantly lower than the 0 and 30 DPI and also was lower than their NCTs, the ITTCs and ITTC-PDSs (*P = 0.001* for all) (Fig. 3A).

The surface temprature of the ITTCs and ITTC-PDSs was signficantly higher at 7, 14 and 20 DPI compared to the ICTs and NCTs (*P = 0.001* for all). However, at 20 DPI, the surface temprature of the ITTCs and ITTC-PDSs signficantly decreased compared to the 14 DPI (*P = 0.001* for both). The surface temprature of the ICTs reached its normal level at 20 DPI but the surface temperature of the treated lesions reached its normal level at 30 DPI (Fig. 3B).

### Ultrasonography

**Figure 4:**
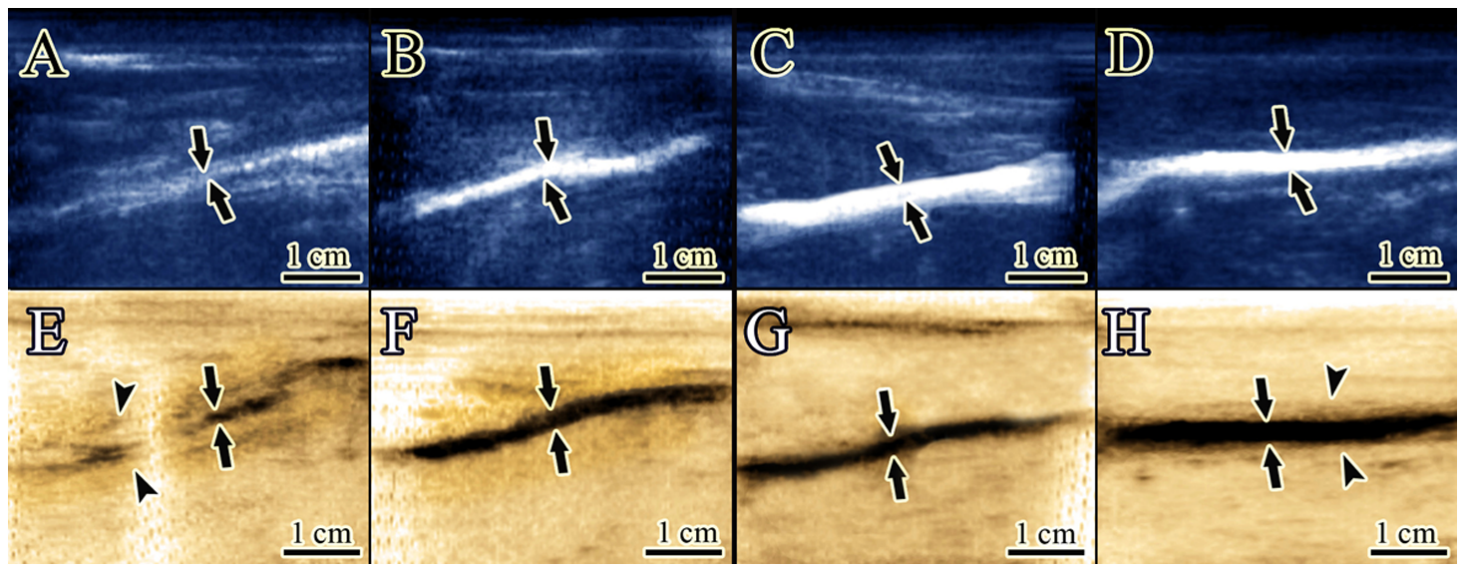
Ultrasonographical characteristics of the tendons. The ICT shows hypoechogenicity with high amount of peritendinous adhesion that is observed as a hypoechoic pattern in the peritendinous area (A). This tendon also has amputated view under inverted ultrasonography (E) that is characterized with irregular hyper echogenicity together with the anechoic pattern (E). The diameter of the regenerated tissue (Arrows) is low (A) and severe development of peritendinous adhesion is evident (E, arrows head). The ITTC, shows hyperechogenicity (B) together with homogeneity (F) of the echogenic area of the tendon (Arrows). The diameter of the new tendon is characteristically higher than ICT (F *vs.* E). ITTC-PDS (Arrows) has higher transverse diameter compared to the ITTC (Arrows) and ICT (Arrows). It is also more echogenic and no amputated pattern is observable in this tendon (C). These tendons (B and C) are more uniform than ICTs. The amount of peritendinous adhesion is also decreased in this tendon (C). Intact tendons have a regular pattern with the hyper echogenic texture (D,H). The paratenon can be seen around the Achilles apparatus as two tiny echogenic lines (D,H Arrows head). In an inverted view better judgment can be made on the differences of the tendons.

Treatment with collagen and collagen-PDS implants significantly improved the scoring number of echogenicity of the ITTCs and ITTC-PDSs compared to that of the ICTs (3 (2–3) ^ICTs^ *vs*. 2 (1–3) ^ITTCs^ *vs*. 2 (0–3) ^ITTC-PDSs^, *P = 0.026*, *P = 0.001*, respectively). In addition, the ITTCs and ITTC-PDSs got better scoring for homogenicity of the injured area compared to that of the ICTs (3 (3–3) ^ICTs^ *vs*. 3 (1–3)^ ITTCs^ *vs*. 3 (1–3)^ ITTC-PDSs^, *P = 0.001* for both). Collagen and collagen-PDS scaffolds resulted in significant reduction of peritendinous adhesion in the ITTCs and ITTC-PDSs compared to that of the ICTs (3 (2–3) ^ICTs^ *vs*. 1.5 (0–3) ^ITTCs^ *vs*. 1 (0–3) ^ITTC-PDSs^, *P = 0.001* for both). Treatment with collagen and collagen-PDS implants significantly increased the regeneration volume of the ITTCs and ITTC-PDSs compared to that of the ICTs (5 (5–5) ^ICTs^ *vs*. 2 (0–2) ^ITTCs^ *vs*. 1 (0–2), *P = 0.001* for both). The ITTCs and ITTC-PDSs had significantly lower peritendinous adhesion compared to that of the ICTs (5 (4–5) ^ICTs^ *vs*. 2 (1–3) ^ITTCs^ *vs*. 1 (0–2) ^ITTC-PDSs^, *P = 0.001* for both). The regenerative proportion scoring of the ITTCs and ITTC-PDSs was significantly improved compared to that of the ICTs (4 (3–5) ^ICTs^ *vs*. 2 (0–2) ^ITTCs^ *vs*. 0 (0–1) ^ITTC-PDSs^, *P = 0.001* for both) (Fig. 4).

### Hematology

Treatment with collagen and collagen PDS implants significantly reduced the leukocyte count in the treated animals compared to the control animals at 10 DPI (*P = 0.049*, *P = 0.025* respectively). At 60 DPI, number of leukocytes in all groups was elevated and approximated the reference range but the measured values were significantly lower in the treated animals compared to the controls (*P = 0.001* for both). At 10 DPI, percentage of the neutrophils of the peripheral blood samples significantly reduced in all groups but the measured values for the treated animals were significantly lower than those of the control animals (*P = 0.001* for both). However, at 60 DPI, percentage of neutrophils increased in all groups and approximated the reference range but the measured value for the treated groups was still significantly lower than that of the control group (*P = 0.001* for both). Percentage of the lymphocytes of the peripheral blood of the animals in all groups was elevated at 10 DPI. At 60 DPI, percentage of the lymphocytes decreased and approximated the normal range but the treated groups had significantly higher percentage of lymphocytes compared to the control group (*P = 0.001*).

Number of platelets in the peripheral blood significantly reduced in all groups, at 10 DPI, but the measured value for the control group was significantly lower than that of the treated groups (*P = 0.001* for both). At 60 DPI, the platelet count of all groups approximated to normal range but the measured value for the control group was significantly lower than that of the treated groups (*P = 0.001* for both) (Table 1).

**Table 1:** Peripheral blood profile of the treated and control animals at 10 and 60 days after the injury.

Time point	10 DPI	10 DPI	10 DPI	60 DPI		60 DPI		60 DPI	
Group	Control (1)	Collagen (2)	PDS (3)	Control (4)		Collagen (5)		PDS (6)	Reference range
	*(n = 5)*	*(n = 5)*	*(n = 5)*	*(n = 20)*		*(n = 20)*		*(n = 20)*	
	*Mean ± SD*	*Mean ± SD*	*Mean ± SD*	*Mean ± SD*		Mean *± SD*		*Mean ± SD*	
**Red blood cells**	5.98±0.92	6.32±0.77	6.55±0.59	7.02±0.68		6.85±0.84		6.98±0.82	5–9×10^12^/L
**Leucocytes**	5.89±0.98	3.8±1.02	3.87±0.87	8.94±0.72		5.14±0.75		4.51±0.72	6–9×10^9^/L
**Neutrophil**	49.82±3.27	42.27±2.99	40.91±3.62	57.71±1.7		45.14±1.95		43.57±1.9	65–70%
**Lymphocyte**	47.82±2.88	51.43±3.33	55.72±4.14	43.26±1.34		49.16±2		54±1.73	30–35%
**Platelet**	98.79±12.39	182.37±33.27	169.47±28.71	153.57±14.03		330.85±26.49		355±31.81	250–400×10^9^/L
			***P value***						
	***1 vs. 2***	***1vs. 3***	***2 vs. 3***	***4 vs. 5***	***4 vs. 6***			***5 vs. 6***	
**Red blood cells**	*0.764*	*0.513*	*0.898*	*0.688*	*0.834*			*0.899*	
**Leucocytes**	*0.049*	*0.025*	*0.277*	*0.001*	*0.001*			*0.447*	
**Neutrophil**	*0.001*	*0.001*	*0.581*	*0.001*	*0.001*			*0.783*	
**Lymphocyte**	*0.089*	*0.049*	*0.385*	*0.001*	*0.001*			*0.001*	
**Platelet**	*0.001*	*0.001*	*0.636*	*0.001*	*0.001*			*0.822*	

DPI, Days Post Injury; SD, Standard Deviation; L, Liter; *vs*, versus. One way ANOVA was performed to statistically analyze the data. Several post hoc Tukey tests were used to statistically analyze the significant differences between groups.

### Serum PDGF Level

Application of collagen and collagen-PDS implants significantly increased the serum PDGF level on the treated animals compared to the control animals, at 60 DPI, so that the PDGF AA level of the collagen and collagen-PDS treated animals were 4.28 and 4.82 fold higher than that of the control animals, respectively (*P = 0.001* for both). The serum PDGF AB level of the collagen and collagen-PDS treated animals were also 2.22 and 2.56 fold higher than that of the control animals, at 60 DPI (*P = 0.001* for both) (Table 2).

**Table 2:** Biochemical findings of the injured tendons at 60 days after the injury.

Time point		60 DPI	60 DPI	60 DPI	*P value*		
Group		Control (1)	Collagen (2)	PDS (3)	*1 vs. 2*	*1 vs. 3*	*2 vs. 3*
		Mean ± SD	Mean ± SD	Mean ± SD			
**PDGF AA (ng/dl)**	***(n = 20)***	1.92±0.93	8.23±1.21	9.26±1.35	*0.001*	*0.001*	*0.228*
**PDGF AB (ng/dl)**	***(n = 20)***	5.32±1.28	11.83±0.58	13.6 3±0.83	*0.001*	*0.001*	*0.044*
**Dry Matter (%)**	***(n = 10)***	11.12±1.21	23.67±1.35	27.76±0.93	*0.001*	*0.001*	*0.001*
**Hydroxyproline (µg/mg dry matter)**	***(n = 10)***	6.85±1.28	28.34±1.81	37.52±1.79	*0.001*	*0.001*	*0.001*

One way ANOVA was performed to statistically analyze the data. Several post hoc Tukey tests were used to statistically analyze the significant differences between groups. Abbreviations: n, Number; SD, Standard Deviation; vs, versus. PDGF, Platelet derived growth factor; DPI, Days Post Injury.

### Gross Pathology

**Figure 5:**
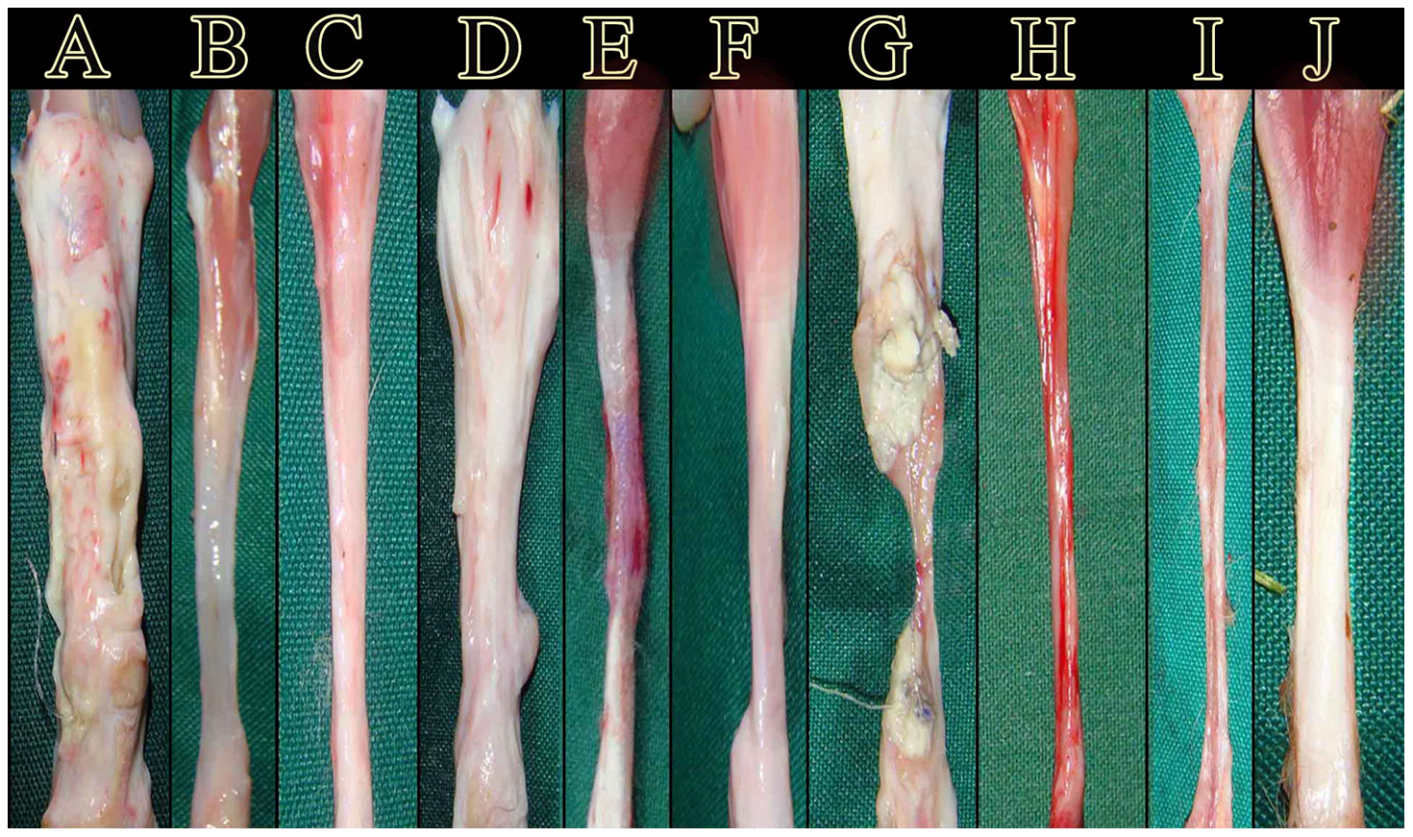
Gross pathological changes in the injured tendons. A–C: ITTC. A: 10, B: 30, C: 60 DPI. D–F: ITTC-PDS. D: 10, E: 30, F: 60 DPI. G–I: ICT. G: 10, H: 30, I: 60 DPI. J: NCT. At 10 DPI, the transverse diameter of the treated lesions is significantly increased compared to the control tendons (A and D vs. G). The implants are surrounded by the newly regenerated fibrous connective tissue at this stage. At 30 DPI, the collagen implant is completely degraded and a new tendon formed (B). The tendon is transparent at this stage (B). Compared to the ITTC, the remnants of the PDS sheath is seen in the injured area of the ITTC-PDSs at 30 DPI (E). Compared to the treated lesions, the control tendon and its gastro soleus muscle are atrophied at this stage and sever hyperemia is seen all over the tendon (B and E vs. H). At 60 DPI, the newly regenerated tendon is matured and is dense. The muscle is less atrophied and less fibrosis is evident in the treated lesions compared to control (C and F vs. I). At 60 DPI, the severity of hyperemia is decreased in the ICTs (I) but it is completely atrophied and its diameter is small. Unlike the ICTs, the transverse diameter of the treated lesions (C and F) is comparable to normal intact tendon (J). Compared to the ITTC, the ITTC-PDS shows less muscle atrophy and no remnants of the PDS sheath is visually evident at gross level (E).

The ITTCs (3 (1–3)) and ITTC-PDSs (2 (1–3)) showed significantly better scores for peritendinous adhesion compared to the ICTs (3 (2–3); *P = 0.049*, *P = 0.021*, respectively). The ITTCs (2 (2–3)) and ITTC-PDSs (2 (0–3)) also showed better scores for hyperemia compared to the ICTs (3(2–3); *P = 0.035*, *P = 0.003*, respectively). The ITTCs and ITTC-PDSs exhibited significantly better scores for muscle fibrosis (3 (1–3) *vs*. 2 (0–3) *vs*. 3 (3–4), *P = 0.042*, *P = 0.001* respectively), muscle atrophy (3 (0–3) *vs*. 2 (1–3) *vs*. 3 (2–4), *P = 0.049*, *P = 0.017* respectively), and general appearance of the injured tendons (2 (0–3) *vs*. 2 (0–4) *vs*. 3 (1–4), *P = 0.044*, *P = 0.035* respectively) compared to the ICTs. The transverse diameter of the injured area of the ICTs was significantly lower than the comparable area in their NCTs (1.89±0.12 mm *vs*. 3.85±0.32 mm, *P = 0.001*). The transverse diameter of the ICTs was also significantly lower than the comparable area of the ITTCs (1.89±0.12^ICTs^ mm *vs*. 4.12±0.28^ITTCs^ mm, *P = 0.001*) and the ITTC-PDSs (1.89 ^ICTs^ ±0.12 mm *vs*. 4.19±0.35 ^ITTC-PDSs^ mm, *P = 0.001*) (Fig. 5).

### Histopathologic Findings

#### General mechanistic report

**Figure 6:**
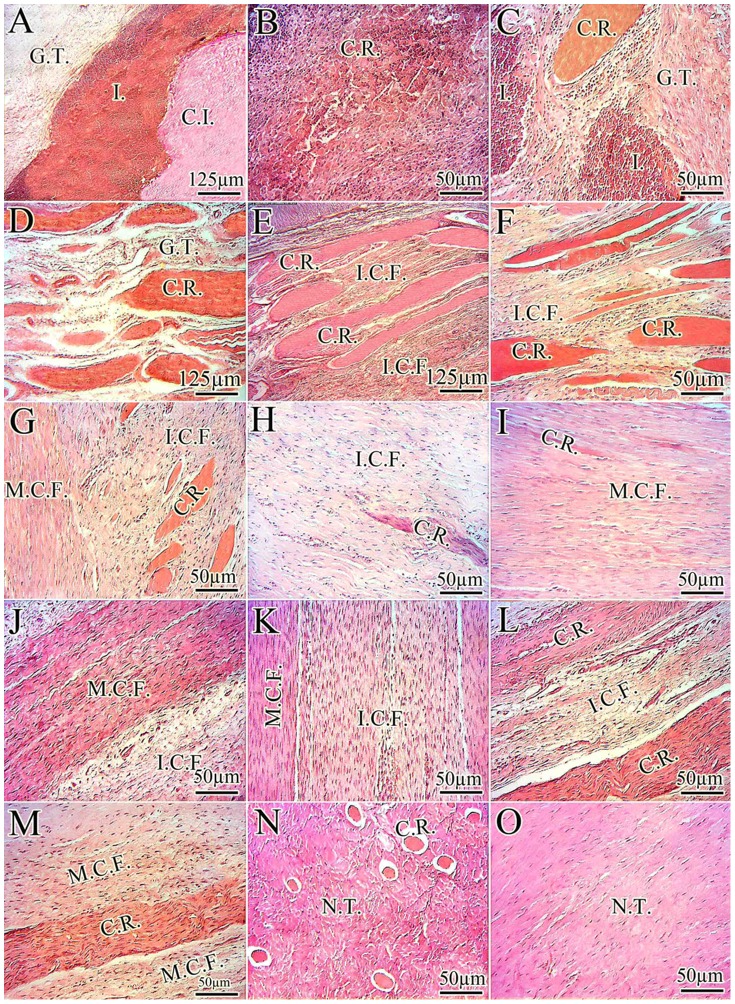
Host implant interaction (Mechanism of collagen implant absorption and its role on tendon healing). At 10 DPI the collagen implant absorbed the inflammatory and mesenchymal cells so that three areas can be observed (A). In the first area, collagen implant (C.I.) is unaffected by the inflammatory cells. The second area is characterized by infiltration of the inflammatory cells (I.) and the third area is a newly developed granulation tissue (G.T.). In the second area, the inflammatory cells degrade the collagen implant and the remnants of the collagen implant are seen (B). At 15 DPI, some parts of the collagen implant are degraded by the inflammatory cells but some parts of the collagen implant are unaffected and are called collagen remnant (C.R.). The G.T. is developed between the degraded parts of the collagen implant. From 20 to 60 DPI, the preserved parts of the collagen implant are incorporated with newly developed healing tissue by three mechanisms. Mechanism 1 (D to I) is a progressive degradation in which the unaffected parts of the collagen implant act as a micro scaffold for the newly regenerated connective tissue and align the new tissue along their orientation. They are gradually degraded but not as fast as those parts degraded at inflammatory phase (B). in this mechanism the collagen implant is almost degraded and replaced by the new tendon so that at 60 DPI (I) the new tendon can be seen. Mechanism 2 (J and K) is the rapid absorption of some parts of the collagen implant. In such mechanism some parts of the collagen implant are absorbed at earlier stages (e.g. 10 DPI) and replaced by the new connective tissue which is matured over time. At later stages (e.g. 30 DPI) some other parts of the collagen implant are absorbed and replaced by the newly developed connective tissue so that at 30 (J) to 40 (K) DPI, two types of newly developed mature and immature collagen fibers (M.C.F. and I.C.F.) can be seen. The M.C.F. act as a micro scaffold for the I.C.F. and align them along their longitudinal direction. These immature collagen fibers are more mature in later stages of tendon healing (J vs. K). Mechanism 3 (L and M) is the graft acceptance in which some preserved parts of the collagen implant are infiltrated by the healing fibroblasts and act as a micro scaffold for the newly developed connective tissue. N: Transverse section of the healing tendons. At 60 DPI, most parts of the collagen implant are degraded and replaced by the new tendon. At longitudinal section (O) the new tendon (N.T) is seen and no remnant of the collagen implant is seen, which means the implant is almost degraded and replaced by the newly aligned tendinous tissue. *Color staining: H&E.*

**Figure 7:**
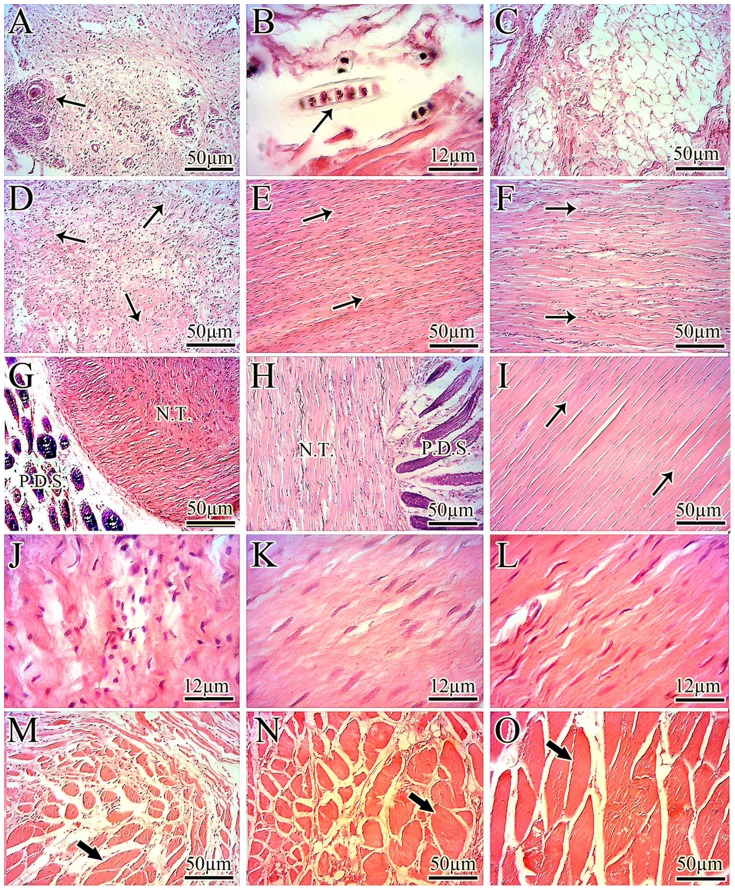
Histologic findings. A: The remnants of the polydioxanone (PDS) suture knots are shown by arrow. B: The arrow shows a remnant of the PDS suture in the healing tissue. C: Injured control tendon (ICT), 60 DPI: fatty tissue has infiltrated in the defect area so that no tendinous tissue is seen. D: An ICT, 60 DPI: the newly developed collagen fibers are immature, and the tissue is amorphous. E: An injured treated tendon with collagen implant (ITTC), 60 DPI: the collagen fibers are mature and highly aligned. F to H: An injured treated tendon with collagen-PDS implant (ITTC-PDSs), 60 DPI: the collagen fibers are aligned and are highly mature. In the transverse (G) and longitudinal (H) sections, the remnants of the PDS scaffold are seen which cover the new tendon (N.T.). No remnant of the collagen implant is seen and all of the tendinous tissue is newly developed. I: A normal intact tendon. The collagen fibers are highly dense and are aligned. In figures D to F and I the double arrows show the direction of the collagen fibers. J: An ICT, 60 DPI: the cells are immature and randomly distributed in the new tissue. The collagen fibers are highly immature and no characteristic alignment can be seen. K: An ITTC, 60 DPI: most of the cells are mature fibroblast and fibrocytes which are laid along the direction of the newly developed highly aligned collagen fibers. L: An ITTC-PDSs, 60 DPI: the majority of the cells are fibrocyte with few number of mature fibroblast. The cells are highly aligned and laid along the direction of the collagen fibers. M: a gastro soleus muscle of the ICT, 60 DPI. Look, the connective tissue is developed between the atrophic muscle fibers which indicates both fibrosis and atrophy of this muscle. N: a gastro soleus muscle of the ITTC, 60 DPI. A lesser amount of connective tissue is developed between the muscle fibers (arrow) and size of the muscle fibers is larger than M which indicates lower degree of muscle fibrosis and atrophy. O: is a normal (intact) gastro soleus muscle. In a normal muscle, the fibers are larger than M and N and less connective tissue is evident between the fibers.

**Figure 8:**
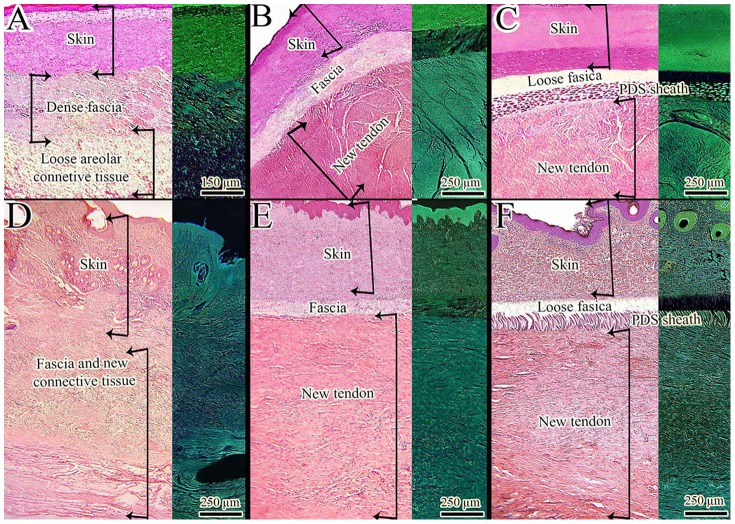
Intrinsic and extrinsic mechanisms of tendon healing. A–C: Transverse section of the skin, fascia and defect area after 60 days post injury (DPI). D–F: Longitudinal section of the skin, fascia and defect area after 60 DPI. A and D: control (defect) tendons. B and E: Injured treated tendons with collagen implant. C and F: Injured treated tendons with collagen-PDS implant. In the control lesions no tendinous structure is formed in the defect area at 60 DPI and the defect is filled with a loose areolar connective tissue. This suggests that the intrinsic mechanism of tendon healing was not effective in producing new tendon in the control group. In the control group a fascia was formed between the skin and defect area and therefore the peritendinous adhesion was well developed. In these tendons, the layers could not be well distinguished from each other and the skin is tightly adhered to the newly regenerated tissue and therefore, these tendons had no function because they cannot move in their subcutaneous space. Implantation of the collagen implant was able to produce a new dense tendinous structure in the defect area and also reduced peritendinous adhesion. These findings suggest that the collagen implant resulted in improving the intrinsic mechanism of tendon healing and was effective in reducing extrinsic mechanism which is responsible for the development of peritendinous adhesions. Therefore, a fascia could be seen between the skin and new tendon but the density and amount of this fascia is lower than those seen in the control lesions at the same stage. Implantation of the collagen-PDS implant significantly reduced peritendinous adhesions as compared to controls and also a new tendon is formed inside the PDS sheath while the collagen implant is replaced by the new tendon. In these tendons no marked peritendinous adhesion is formed around the new tendon but a loose fascia is present just under the skin which is normal. The remnants of the PDS scaffold are present at the periphery of the newly regenerated tendon. These findings suggest that the PDS sheath completely inhibited the extrinsic mechanism of tendon healing and tendon adhesions but had no deleterious effect on the intrinsic mechanism of tendon healing. The right one/third of the figures are inverted to differentiate the structures more comprehensively.

Inflammation was not characteristically present in the injured area of the ICTs at 10 DPI as it was seen in the ITTCs and ITTC-PDSs. However, application of collagen and collagen-PDS implants in the defect area increased the inflammation markedly so that at 10 DPI, the inflammatory cells infiltrated into the implants. At this stage, three different areas could be distinguished in the injured area of the treated tendons including: some areas of the implant which were not affected by the inflammatory cells, a demarcation area between the implant and the newly regenerated granulation tissue, in which mostly neutrophils and macrophages were predominant. In the treated group by collagen implant, the newly regenerated fibrous connective tissue was originated from both the intrinsic and extrinsic tendon healing, which means that the granulation tissue covered the implant at the periphery but the granulation tissue was also originated from the tendon ends. In the treated lesions with collagen-PDS implant the extrinsic mechanism of tendon healing was inhibited by the PDS scaffold and the granulation tissue was mostly originated from the intrinsic mechanism of tendon healing (between the tendon ends). The inflammation partially degraded the collagen implant in both of the treated groups, at 10 DPI, and the newly regenerated connective tissue filled the free spaces of the implant. Some parts of the collagen implants were not degraded and were preserved at 10 DPI. During 15–60 DPI, the remnants of the collagen implant gradually degraded but also collaborated in tendon remodeling at later stages of tendon healing in which they acted as scaffolds for the newly regenerated tendinous tissue and aligned the new tissue along their longitudinal directions. These micro scaffolds were gradually absorbed at later stages of healing or were infiltrated by the fibroblasts and were accepted as a part of the new tendon. There was also another mechanism in which those degraded parts of the implant that were filled with the granulation tissue at 10 DPI were matured at further stages of tendon healing (e.g. 30–60 DPI) so that they also acted as scaffolds for the newly regenerated connective tissue that replaced the other parts of the collagen implant at fibroplasia or remodeling stages of tendon healing. In addition, the PDS part of the scaffold acted as a macro scaffold for the newly regenerated tendinous tissue and had a role in alignment of the newly regenerated tendinous tissue in the defect area. The PDS sheath was gradually absorbed but its remnants were still present at 60 DPI and covered the periphery of the new tendon. The PDS suture also degraded partly so that the suture strands showed no continuity at 60 DPI but their remnants were still present at that time (Fig. 6–8).

#### Quantitative and semi quantitative reports

The ICTs had significantly higher cellularity, total fibroblast, immature fibroblast and lymphocyte, compared to the ITTCs and ITTC-PDSs (*P = 0.001* for all), at 60 DPI. The ICTs also had significantly lower number of mature fibroblast, fibrocyte, neutrophil, blood vessel, diameter of fibrocyte, small, medium and large blood vessels, and collagen density compared to the ITTCs and ITTC-PDSs (*P<0.05* for all). At this stage, the ITTC-PDSs had significantly higher number of total cells, fibrocyte, neutrophil, lymphocyte, plasma cell, macrophage, large-sized blood vessels and collagen and cell densities and had significantly lower number of total fibroblast and total vessels compared to the ITTCs (*P = 0.001* for all). The ITTC-PDSs and ITTCs had significantly higher number of total cell, total fibroblast, immature and mature fibroblast and had significantly lower number of fibrocyte compared to the normal uninjured tendons (*P<0.05* for all) (Table 3).

**Table 3:** Histopathological findings (number and diameter of different cellular and vascular structures in the histopathologic field).

Time point	60 DPI	60 DPI	60 DPI						
Group	Control	Collagen	Collagen-PDS	Normal	*P value*	*P value*	*P value*	*P value*	*P value*
	(1)	(2)	(3)	(4)	*1 vs. 2*	*1 vs. 3*	*2 vs. 3*	*2 vs. 4*	*3 vs. 4*
	(n = 250 HF)	(n = 250 HF)	(n = 250 HF)	(n = 250 HF)					
	Mean ± SD	Mean ± SD	Mean ± SD	Mean ± SD					
**Total Cell ** ***(n)***	523.41±19.14	373.44±8.01	401.2±9.59	52.38±4.18	*0.001*	*0.001*	*0.001*	*0.001*	*0.001*
**Total Fib ** ***(n)***	334.24±10.9	257.06±5.31	241.17±9.1	52.38±4.18	*0.001*	*0.001*	*0.042*	*0.001*	*0.001*
**Immature Fib ** ***(n)***	221.82±10.83	96.41±5.63	85.70±5.07	4.12±0.92	*0.001*	*0.001*	*0.098*	*0.001*	*0.001*
**Mature Fib ** ***(n)***	111.44±6.43	144.68±8.99	131.82±6.11	12.89±2.85	*0.001*	*0.001*	*0.126*	*0.001*	*0.001*
**Fibrocyte ** ***(n)***	3.72±1.64	17.15±2.64	24.94±3.28	35.71±5.79	*0.001*	*0.001*	*0.037*	*0.001*	*0.012*
**Neutrophil ** ***(n)***	7.29±1.73	14.18±2.04	23.47±3.66		*0.001*	*0.001*	*0.001*		
**Lymphocyte ** ***(n)***	87.31±7.4	51.16±4.3	66.44±5.31		*0.001*	*0.001*	*0.001*		
**Plasma cell ** ***(n)***	4.19±1.54	2.67±1.3	12.54±2.24		*0.271*	*0.001*	*0.001*		
**Macrophage ** ***(n)***	59.32±8.19	43.23±3.89	53.67±3.19		*0.001*	*0.001*	*0.387*		
**Vessels ** ***(n*** **)**	12.35±2.96	33.44±3.93	25.19±3.65		*0.001*	*0.001*	*0.033*		
**Small Vessels ** ***(n)***	7.84±0.63	26.7±2.02	19.88±1.29		*0.001*	*0.001*	*0.001*		
**Medium Vessels ** ***(n)***	3.07±0.88	3.67±0.7	5.17±1.01		*0.823*	*0.401*	*0.485*		
**Large Vessels ** ***(n)***	0.62±0.56	3.09±0.98	5.05±1.39		*0.001*	*0.001*	*0.372*		
**Immature Fib ** ***(D) µm***	7.93±1.23	6.72±0.87	6.29±0.43		*0.227*	*0.382*	*0.923*		
**Mature Fib ** ***(D) µm***	3.7±0.15	3.35±0.18	3.57±0.14		*0.176*	*0.644*	*0.510*		
**Fibrocyte ** ***(D) µm***	1.69±0.04	1.32±0.04	1.3±0.03		*0.001*	*0.001*	*0.462*		
**Small Vessels ** ***(D) µm***	11.9±0.86	15.64±1.03	15.36±0.82		*0.001*	*0.001*	*0.922*		
**Medium Vessels ** ***(D) µm***	34.41±1.58	42.9±2.65	45.29±2.24		*0.001*	*0.001*	*0.198*		
**Large Vessels ** ***(D) µm***	69.19±4.08	82.47±3.68	92.6±3.61		*0.001*	*0.001*	*0.001*		
**Collagen Density (%)**	8.21±1.86	35.19±3.3	45.08±1.98	94.28±2.17	*0.001*	*0.001*	*0.001*	*0.001*	*0.001*
**Cell Density (%)**	24.77±3.4	15.81±0.98	21.49±2.32	3.21±0.32	*0.001*	*0.309*	*0.001*	*0.001*	*0.001*
**Background Density (%)**	63.16±2.27	49.63±2.98	35.4±2.16	2.39±1.24	*0.001*	*0.001*	*0.001*	*0.001*	*0.001*

One way ANOVA was performed to statistically analyze the data. Several post hoc Tukey tests were used to statistically analyze the significant differences between groups. Data were expressed as Mean and Standard deviation. Abbreviations: HF, Histopathologic field; D, Diameter; µm, Micrometer; n, Number; SD, Standard Deviation; vs, versus.

The ITTC-PDSs and ITTCs showed significantly better scoring for the collagen fiber alignment (0 (0–2) ^ITTC-PDSs^ vs. 1 (1–3)^ ITTCs^ *vs*. 3 (2–3)^ ICTs^, *P = 0.001* for both), perivascular edema (0 (0–1)^ ITTC-PDSs^ *vs*. 0.5 (0–1)^ ITTCs^ *vs*. 2 (2–3)^ ICTs^, *P = 0.001*, *P = 0.012* respectively), tissue maturity (1.5 (0–2)^ ITTC-PDSs^ *vs*. 1.5 (1–2)^ ITTCs^ *vs*. 3.5 (2–4)^ ICTs^, *P = 0.001*, *P = 0.044* respectively), crimp pattern (1.5 (1–2)^ ITTC-PDSs^ *vs*. 1.5 (1–2)^ ITTCs^ *vs*. 4 (4–4)^ ICTs^, *P = 0.001* for both) and vascularity (1 (1–2)^ ITTC-PDSs^ *vs*. 1.5 (1–4)^ ITTCs^ *vs*. 4.5 (4–5)^ ICTs^, *P = 0.001* for both) compared to the ICTs (Fig. 7).

### Scanning Electron Microscopy

**Figure 9:**
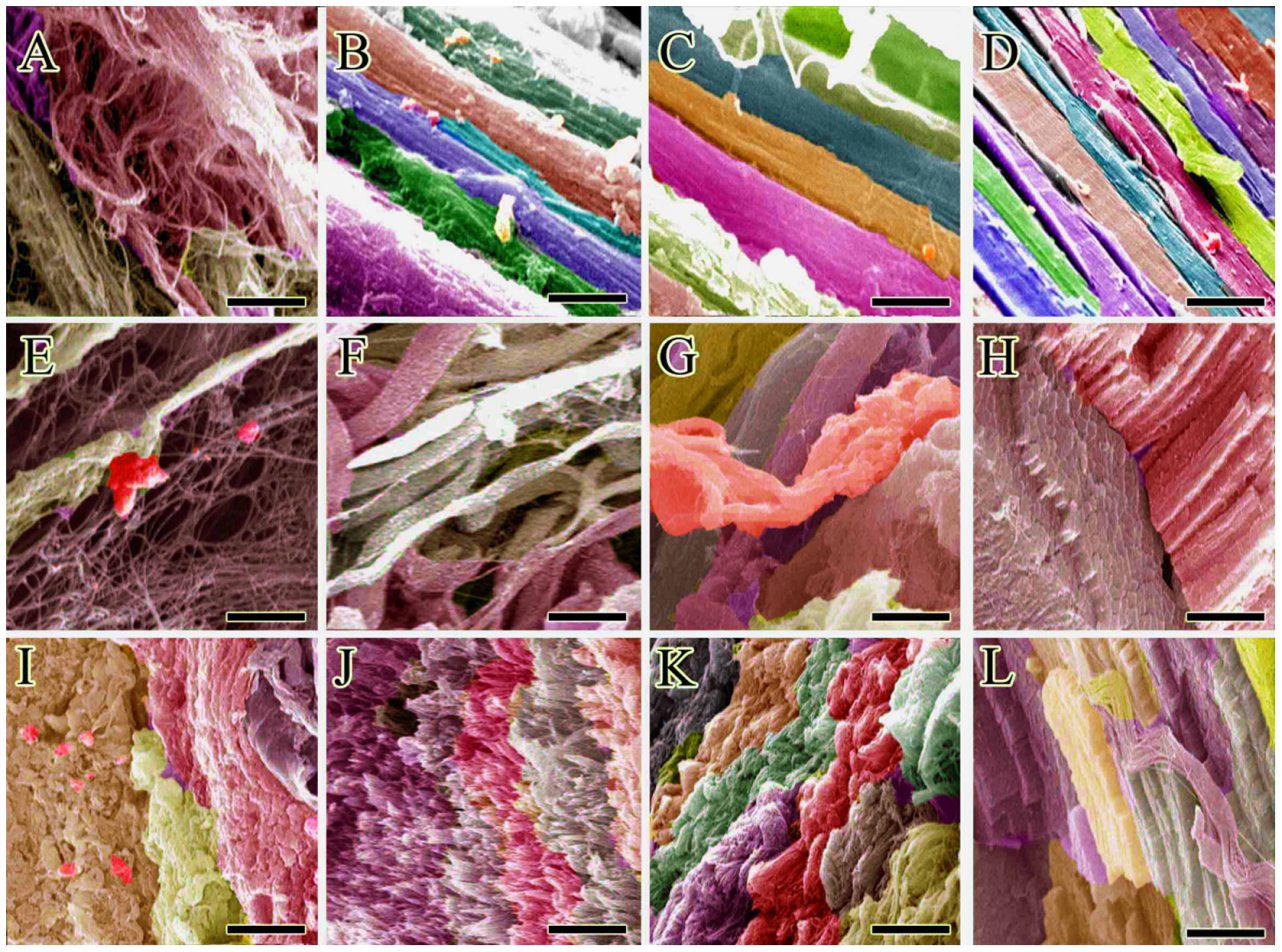
Ultrastructural characteristics of the tendons (powered by colored scanning electron microscopy). ICTs had a lower density of the collagen fibrils (A) and aggregation of this haphazardly oriented collagen fibrils failed to produce the collagen fibers as it was seen in the ITTCs (B), ITTC-PDSs (C) and Intact tendons (D). Compared to the ITTCs (B) the fibers were more differentiated and developed in the ITTC-PDSs (C). At a larger magnification (E–H), the collagen fibrils were randomly distributed in the ICTs and not aggregated as fibers. The diameter and density of the collagen fibrils was low. Unlike ICTs, the fibers were more developed in the treated lesions (F, G) but these fibers were not highly aligned as it can be seen in intact tendons (H). I–L: are the transverse sections of the tendons. In the ICTs the cellularity was high and no diagnostic fibers and fiber bundles formed. The section was only filled with the unorganized collagen fibrils that produce an amorphous structure at lower magnification (I). Fibers and Fiber bundles were differentiated in the ITTCs (J) and ITTC-PDSs (K) and could be distinguished from each other. These fibers compactly aggregated to produce the bundles of fibers. Although these fibers were formed they were not as dense as those we have seen in intact tendons (L). This is because the density and compactness of the collagen fibrils of the ITTCs and ITTC-PDSs were lower than intact tendons. *Scale bar: A = 5 µm, B–D = 40 µm, E–G = 15 µm, H = 80 µm, I–L = 25 µm*.

Treatment with collagen-PDS implant significantly increased the diameter of the collagen fibrils compared to those of the ITTCs and ICTs (*P = 0.001* for both). Treatment with collagen implant also significantly increased the diameter of the collagen fibrils compared to that of the ICTs (*P = 0.001*). The collagen fibers of the ITTC-PDSs had significantly higher transverse diameter compared to that of the ITTCs (*P = 0.001*). The ITTCs and ITTC-PDSs had significantly lower cellularity compared to that of the ICTs (*P = 0.001*), at 60 DPI, but there were no significant differences between the cellularity of the ITTCs and the ITTC-PDSs at this stage (*P = 0.081*). Treatment with collagen-PDS implant significantly increased the density of the collagen fibrils compared to those of the ITTCs and ICTs (*P = 0.049* and *P = 0.001*, respectively). The ITTCs also had significantly higher collagen fibril density compared to that of the ICTs (*P = 0.001*). The ITTC-PDSs had significantly lower cell density compared to that of the ICTs (*P = 0.049*) but there were no significant differences between cell density of the ITTCs and ICTs at 60 DPI (*P = 0.109*) (Table 4). Treatment with collagen and collagen-PDS implants resulted in aggregation of collagen fibrils into collagen fibers and fiber bundles but in the unassisted control lesions the collagen fibrils failed to differentiate into fibers. Treatment with collagen-PDS implant significantly improved alignment of collagen fibrils compared to those of the ITTCs and ICTs (2 (0–2)^ITTC-PDSs^ *vs*. 2.5(0–3)^ITTCs^ *vs*. 3(3–4)^ICTs^, *P = 0.023*, *P = 0.001*, respectively). The ITTC-PDSs also gained significantly better scoring for maturation of the collagen fibrils than those of the ITTCs and ICTs (1(1–2) *vs*. 2 (2–3) *vs*. 4 (3–4), *P = 0.012*, *P = 0.001* respectively). There were no significant differences between the crimp pattern of the ITTCs and ITTC-PDSs (*P = 0.281*) but both gained significantly better scoring for crimp pattern compared to that of the ICTs (2 (2–3)^ITTC-PDSs^ *vs*. 2(2–4)^ITTCs^ *vs*. 4 (4–4)^ICTs^, *P = 0.001* for both) (Fig. 9).

**Table 4:** Morphological characteristics of the injured tendons at 60 days after injury (Scanning electron microscopy).

Time point	60 DPI	60 DPI	60 DPI	*P value*	*P value*	*P value*
Group	Control (1)	Collagen (2)	PDS (3)	*1 vs. 2*	*1vs. 3*	*2 vs. 3*
	Mean ± SD	Mean ± SD	Mean ± SD			
**Fibrils (D)**	29.52±3.27	46.21±4.15	57.76±3.88	*0.001*	*0.001*	*0.001*
**Fibers (D)**	–	456.84±14.46	512.01±17.95	*0.001*	*0.001*	*0.001*
**Cells (n)**	257.33±11.63	187.54±7.55	172.53±9.04	*0.001*	*0.001*	*0.081*
**Fibrils’ Density (%)**	33.24±4.43	58.88±3.81	65.1±2.35	*0.001*	*0.001*	*0.049*
**Cell Density (%)**	22±3.3	18.86±1.65	16.45±1.44	*0.109*	*0.049*	*0.199*

One way ANOVA was performed to statistically analyze the data. Several post hoc Tukey tests were used to statistically analyze the significant differences between groups. Number of tissue samples: n = 10, number of tissue sections: n = 5, number of scanning electron microscopic ultramicrographs for each tissue sections in each analysis: n = 5 (totally = 250 ultramicrographs for each group). Abbreviations: D; diameter, n; number.

### Dry Matter Content

Treatment with collagen and Collagen-PDS implants significantly increased the dry matter content of the ITTCs and ITTC-PDSs at 60 DPI compared to the ICTs (27.76±0.93^ITTC-PDSs^ % *vs*. 23.67±1.35^ITTCs^ % *vs*. 11.12±1.21^ICTs^ %, *P = 0.001* for all) (Table 2).

### Hydroxyproline

The hydroxyproline content of the ITTC-PDSs was significantly higher than that of the ITTCs (*P = 0.001*) at 60 DPI and both the ITTC-PDSs and ITTCs had significantly higher hydroxyproline content compared to the ICTs (37.52±1.79 ng/µg ^ITTC-PDSs^ *vs*. 28.34±1.81 ng/µg ^ITTCs^ *vs*. 6.85±1.28 ng/µg ^ICTs^, *P = 0.001* for both) (Table 2).

### Biomechanical Findings

At 60 DPI, treatment with collagen-PDS implant significantly increased the maximum load (1.4 fold), yield load (1.5 fold), stiffness (1.46 fold), maximum stress (1.43 fold) and modulus of elasticity (1.63 fold) in the ITTC-PDSs compared to those of the ITTCs (*P = 0.001* for all) and both of the ITTCs and ITTC-PDSs had significantly higher maximum load (5.36 and 7.53 fold, respectively), yield load (5.23 and 7.87 fold, respectively), stiffness (8.81 and 12.93 fold, respectively), and modulus of elasticity (2.37 and 3.87 fold, respectively) compared to those of the ICTs (*P = 0.001* for all). There were no significant differences in the ultimate strain and yield strain between the ITTCs and ITTC-PDSs and both had significantly lower ultimate strain (0.62 and 0.57 fold, respectively) and yield strain (0.67 and 0.62 fold, respectively) compared to that of the ICTs (*P = 0.001* for all). Treatment with collagen and collagen-PDS implants significantly increased the cross sectional area (measured as an ellipse) compared to that of the ICTs (*P = 0.001* for both). There were no significant differences between the biomechanical properties of the intact tendons of all groups. The biomechanical properties of the ICTs were significantly lower than those of their intact NCTs (*P = 0.001* for all). Although treatment with collagen and collagen-PDS implants significantly improved the biomechanical properties of the injured tendons compared to that of the ICTs, however their biomechanical properties were still inferior to the biomechanical properties of their intact NCTs at 60 DPI (*P = 0.001* for all) (Table 5).

**Table 5:** Biomechanical properties of the injured and normal contralateral tendons after 60 days of tendon injury.

Variables (Unit)	60 DPI		60 DPI		60 DPI	
	Control (1)		Collagen (2)		Collagen-PDS (3)	
	L	R	L	R	L	R
	(*n = 10*)	(*n = 10*)	(*n = 10*)	(*n = 10*)	(*n = 10*)	(*n = 10*)
	Mean ± SD	Mean ± SD	Mean ± SD	Mean ± SD	Mean ± SD	Mean ± SD
**Maximum load (N)**	9.82±3.91	359.12±41.06	52.72±2.99	346.12±41.39	74.02±7.84	340.12±33.19
**Yield load (N)**	8.05±3.56	309.79±35.78	42.17±6.03	295.89±39.16	63.4±10.5	296.8±34.31
**Stiffness (N/mm)**	2.31±0.54	215.2±19.89	20.37±2.03	205.77±13.52	29.87±3.8	213.21±22.38
**Maximum Strain %**	26.33±2.21	10.66±0.65	16.35±1.15	10.57±0.63	15.07±0.88	10.86±1.04
**Yield strain %**	20.45±2.3	8.33±0.85	13.82±0.91	8.08±0.83	12.84±0.71	8.2±0.65
**Cross section area (mm^2^)**	4.41±0.25	10.31±0.5	16.47±0.76	10.1±0.41	16.17±1.16	10.48±0.62
**Maximum stress (N/mm^2^)**	2.25±0.99	34.8±3.93	3.2±0.24	34.34±4.63	4.6±0.66	32.54±3.74
**Modulus of elasticity (N/mm^2^)**	0.08±0.03	3.28±0.48	0.19±0.01	3.25±0.5	0.31±0.07	3.02±0.52
	***P Value***	***P Value***	***P Value***	***P Value***	***P Value***	***P Value***
	***1 L vs. 2 L***	***1 L vs. 3 L***	***2 L vs. 3 L***	***1 L vs. 1 R***	***2 L vs. 2 R***	***3 L vs. 3 R***
**Maximum load**	0.001	0.001	0.001	0.001	0.001	0.001
**Yield load**	0.001	0.001	0.001	0.001	0.001	0.001
**Stiffness**	0.001	0.001	0.001	0.001	0.001	0.001
**Maximum Strain**	0.001	0.001	0.712	0.001	0.001	0.001
**Yield strain**	0.001	0.001	0.502	0.001	0.001	0.001
**Cross section area**	0.001	0.001	0.854	0.001	0.001	0.001
**Maximum stress**	0.428	0.001	0.001	0.001	0.001	0.001
**Modulus of elasticity**	0.001	0.001	0.001	0.001	0.001	0.001

One way ANOVA was performed to statistically analyze the data. Several post hoc Tukey tests were used to statistically analyze the significant differences between groups. Control L: Injured control tendon; Control R: Normal contralateral tendon (NCT) of the control group. Collagen L: Injured treated tendon by collagen implant. Collagen R: NCT of the Collagen group. PDS L: Injured treated tendon by Collagen-polydioxanone implant. PDS R: NCT of the Collagen-PDS group.

## Discussion

The results of the present study showed: 1) The scaffolds were biocompatible and biodegradable 2) The collagen scaffold was not acutely rejected but also incorporated with the healing tissue 3) The alignment of the collagen implant had a significant role in aligning the newly regenerated collagen fibers and cells 4) Both the collagen implant and PDS sheath increased the inflammation but the collagen implant had a major role in this regard 5) Due to this inflammation the development of the newly regenerated connective tissue was accelerated so that the new tissue filled the free spaces of the collagen implant after the inflammatory cells degraded such parts 6) Both the collagen and collagen-PDS implants had significant roles in reducing the peritendinous adhesion; however the collagen implant by its alignment effect reduced the peritendinous adhesion during tendon healing while the PDS sheath inhibited the extrinsic mechanism of tendon healing in which proliferation of the peritendinous fibroblasts were inhibited by the PDS sheath and the tendon healing was continued by the intrinsic mechanism 7) At 60 DPI, the collagen scaffold was replaced by the newly formed dense tendinous connective tissue 8) The treatment strategy significantly increased the collagen quantity, quality and biomechanical characteristics of the newly developed tendon in the defect area of the treated animals compared to the control ones 9) The implants affected tendon healing by accelerating intrinsic mechanism of tendon healing because they established the continuity of the injured area and provided an optimum environment for cell proliferation, differentiation and maturation 10) Such treatment strategy was able to at least improve the clinical function of the animals.

After application of the bioimplants in the defect area, the continuity of the Achilles apparatus was immediately established. In addition, both of the collagen scaffold and PDS sheath attracted the inflammatory cells in the injured area and this accelerated tendon healing. To prove the above statement, we measured the diameter of the injured area at various time points and showed that the treated lesions had significantly greater diameter compared to the control tendons during the first three weeks following surgery. In addition, the surface temperature of the treated lesions was significantly higher than the controls. We have previously showed that there is a strong correlation between the tendon diameter and the surface temperature with the degree of postsurgical inflammation [12], [39], [42]. These findings were supported by the histological and hematological results, in which we showed that the inflammatory cells infiltrated through the implants and their numbers were reduced in the peripheral blood profile. Unlike the treated lesions, we didn’t see any marked inflammation in the ICTs during the first two weeks after surgery. The role of inflammation in tendon healing should be highlighted [5], [12], [17], [20]. After tendon injury neutrophils, lymphocytes and macrophages infiltrate in the injured area [12], [45]. The neutrophils are predominant in the acute stage of inflammation [18], [39]. These cells, together with the macrophages and the injured cells of the remaining tendon edges and platelets deliver growth factors, cytokines and chemo-attractants which regulate mesenchymal cell (fibroblasts) migration, proliferation and differentiation [5], [12], [40]. The neutrophils are the main cells in the earlier stages of tendon inflammation which have a role in phagocytosis of the collagen implant, while the macrophages have a greater role in delivering cytokines and growth factors and a specific role in facilitating the transition between inflammatory phase of tendon healing to the fibroplasia stage [12], [27], [30], [32], [44]. Macrophages are regulated by the lymphocytes [12], [17], [18], [20].

The collagen molecules have immunogenic activity for the inflammatory cells and their mechanism of absorption is mainly by phagocytosis [12], [27], [30], [32], [44]. However, the absorption mechanism of the PDS sheath is quite different from the collagen implant [12], [31], [33]. The PDS sheath is absorbed via a hydrolysis mechanism and therefore its absorption rate is slower than the collagen implant [27], [33]. Although the PDS sheath has lower immunogenic activity compared to the collagen implant, a combination of collagen implant and PDS sheath has been found to be more immunogenic than the collagen implant alone [12], [27], [33]. Therefore, compared to the control group in which the inflammatory response was not markedly evident at the inflammatory phase of tendon healing, the implants were able to increase the inflammation in the injured area, and for this reason, higher infiltration of the inflammatory cells in the injured treated tendons improved development of granulation tissue in the defect area.

At this time, both the intrinsic and extrinsic mechanism of tendon healing were activated [5], [12]. In the extrinsic mechanism, the fibroblasts originate from the peritendinous tissues which have a role in development of peritendinous adhesion [5], [26] while in the intrinsic mechanism, the healing fibroblasts originate from the tendon edges, and have a major role in tendon healing [5], [26], [46]. In the ICTs, both the intrinsic and extrinsic mechanisms were activated but the response was poor, which means no effective proliferation of fibroblasts was seen in the fibroplasia stage of tendon healing. In the ITTCs, the collagen implant activated both the intrinsic and extrinsic mechanisms of tendon healing, and regarding the effects of collagen implant in increasing the inflammatory response, it was able to accelerate the quality of fibroplasia and resulted in optimum proliferation of fibroblasts in the fibroplasia stages. More fibroplasia response was evident in the ITTC-PDSs; however the PDS sheath inhibited the extrinsic mechanism of tendon healing in which the most effective response was originated from the intrinsic mechanism. Regarding the above explanations, it is clear why the ITTC-PDSs had lower peritendinous adhesion compared to the ITTCs and ICTs.

Interestingly, the amount of peritendinous adhesion in the ITTCs was also significantly lower than the ICTs which means the collagen implant, alone, had a role in reducing the peritendinous adhesion. The role of alignment effect of the collagen implant and its architecture should be highlighted. Due to the three-dimensional architecture of the collagen implant, it absorbed the inflammatory cells in its architecture. These cells partially degraded the collagen implant, thus the free spaces were produced in the collagen implant and the fibroblasts migrated into these free spaces. The fibroblasts or tenoblasts laid along the direction of the collagen fibers of the implant and proliferated and deposited the newly regenerated collagen fibers. Therefore, the newly developed connective tissue was only placed in the defect area because the collagen implant did not allow the healing fibroblasts to proliferate in different directions. This concentrated the healing fibroblasts only in the defect area and inhibited development of peritendinous adhesion and muscle fibrosis.

Unlike the treated lesions, the fibroplastic response, although poor in the ICTs, due to the unorganized newly regenerated connective tissue in the defect area, the healing fibroblasts proliferated haphazardly in various directions including in the peritendinous fascia and gastro soleus muscle. This resulted in collagen deposition not only in the defect area but also resulted in developing the peritendinous adhesion and muscle fibrosis. Development of peritendinous adhesion is one of the major limitations of tendon injury and has major clinical relevance [5], [12], [26], [39].

Less adhesion, and better quality of fibroplasia, increased the collagen content of the injured area which was initially diagnosed at gross, micro and nano morphology, but then it was confirmed by determination of dry matter and hydroxyproline content. Both the collagen implant and the PDS sheath had significant roles in aligning the newly regenerated tissue. Saeidi et al [47] showed that using a disorganized scaffold as a guide to produce highly organized tissue has the potential to delay the production of useful matrix or prevent uniform remodeling. Liu et al [48] showed that the alignment of the electrospun fibrous scaffold has a significant role in cellular alignment *in* *vitro*. Cardwell et al [22] also suggested that large fibers are more effective than aligned fibers in cell behavior *in* *vitro*. Our implant, although aligned in nature, also had large fibers’ diameter. While the collagen implant was gradually degraded and its remnants acted as micro scaffolds for the new tendon, the PDS sheath was degraded more slowly than the collagen implant, so that it acted as macro scaffold for the new tendon and resulted in growth of the healing tissue along the direction of the PDS sheath, which was in accordance to the normal anatomical direction of the Achilles tendon. The ultrasonographical results approved the above findings, in which the treated tendons had less adhesion, and also their echogenicity and homogenicity were closer to the intact tendons compared to the ICTs. The results of the ultrasonography has major clinical value when the results are to be translated in clinical setting [12], [39], [45].

Less adhesion, higher collagen content, more matured fibroblasts and fibrocytes, together with higher dry matter content and advanced alignment of the collagen fibrils and fibers, were well responsible for the significantly improved maximum load, yield load, stiffness and strength of the injured treated tendons compared to the ICTs. This newly regenerated tendinous tissue was effective to restore the function of the animals, so that better scoring for the muscle atrophy together with better physical activity and weight bearing capacity of the animals well proved the above-mentioned statements.

Our gross, micro and nano morphologic results showed that the healing regenerated tendons treated with the bioimplants were in the remodeling stage of tendon healing in which the ITTCs were in the early remodeling while the ITTC-PDSs were in the mid remodeling phases of tendon healing [5]. In fact, the collagen implant had a major role in tendon healing while the PDS sheath had an ancillary role in tissue alignment and maturation but its role should not be neglected. The morphologic results were confirmed by the serum PDGF level. Positive correlation between the PDGF level and quality of the healing response and higher values of hydroxyproline has previously been reported [49]. Hee et al [49] showed that this growth factor is chemotactic, mitogenic, and pro-angiogenic. It regulates proliferation of tendon cells and motivates extracellular matrix deposition and organization. It also improves the morphological and biomechanical properties of healing tendons and ligaments. Work by Duffy et al. [50] has shown that PDGF is elevated in the healing canine digital flexor tendon, suggesting its role in the healing process. Solchaga et al [51], in a cohort study in humans, also showed an increase in the serum PDGF level after implantation of Augment (®) Bone Graft.

After producing tendon injury the number of platelets decreased in the peripheral blood of all the groups. Thrombocytopenia has been stated as a usual outcome after many orthopedic surgeries [52]–[54]. Compared to the controls, the treated animals had higher number of platelets in their peripheral blood both at 10 and 60 DPI. In addition, the platelet number of the peripheral blood was higher at 60 DPI than that of the 10 DPI in all the groups. It seems the implants acted as a hemostatic agent. Following injury induction hemorrhage had occurred as the result of disruption of the regional vessels. Possibly, the implants absorbed the blood platelets in their internal architecture. It seems the absorbed platelets in the implants were preserved and protected from the access of inflammatory phagocytic cells during the inflammatory phase of tendon healing and thus, those platelets which were present in the most internal architecture of the implants had minimum exposure to the inflammatory cells. Therefore, their α-granules had this opportunity to gradually release their PDGF during tendon healing and this could be one reason that the treated animals had higher serum PDGF level at 60 DPI than the control animals. In addition to platelets, PDGF has been shown to be secreted by activated macrophages and endothelial cells [55]. Based on our findings, the implants accelerated the inflammatory response for a short period at the earlier stages of tendon healing and during this phase the activated macrophages possibly had a role in secreting this growth factor. Unlike treated lesions, in the control lesions, due to absence of the implant in the defect area, the aggregated platelets were in the access of inflammatory cells and were possibly removed faster from the injured area, by these phagocytic cells and therefore these platelets had lower chance to release their PDGF from their α-granules.

PDGF plays a role in the angiogenesis [56]. Our histologic evaluations suggest that the treated tendons had parallel blood vessels along the aligned collagen fibers and the vessels were more mature than those of the control lesions which were haphazardly distributed in the injured area. Application of the bioimplants in the defect area, resulted in less hemorrhages and therefore, less platelets had spent in the injured area. Unlike the treated lesions, the vessels of the control lesions were less mature and were more unorganized and therefore it is possible that they were disrupted during the physical movement of the animals during tendon healing because of their delicate wall and unorganized distribution. This disruption caused micro hemorrhages and therefore higher number of platelets had spent in the injured area which resulted in decreased platelet number in the peripheral blood of the control animals. Our gross morphologic results confirmed the above theory and showed that the regenerated loose tissue in the control animals was hyperemic even after 60 DPI. This suggests that occurrence of micro-hemorrhages was still in progress in the control animals. We observed less hyperemia in the treated lesions. Thus one possible mechanism is the anti-hemorrhagic effects of this treatment strategy which may explain why higher platelet number was present in the peripheral blood of the treated animals compared to the control ones. It seems, implantation of the collagen and collagen-PDS implants may had a role in increasing the serum PDGF level and the higher serum PDGF level observed in the treated animals had positive correlation with the higher number of platelets in the peripheral blood of these animals. However, this should be further investigated and needs more confirmation. Despite of these explanations, PDGF is an indicator of the quality of tendon healing and the higher serum PDGF level observed in the treated animals suggest a better quality of tendon healing as compared to the control animals.

At gross level, the implants were not detectable at 60 DPI and the scaffolds were replaced by the new tendon which had significant similarities to the intact tendon. At this stage the unassisted tendon healing in the ICTs failed to produce a new dense connective tissue in the defect area and the only regenerated tissue was a loose areolar connective tissue which was infiltrated by fatty tissue. Our histologic results showed that although the healing ICTs were not in the inflammatory phase of tendon healing, their healing characteristics did not match any described phases of tendon healing, suggesting no effective healing response had occurred in these tendons [5], [12]. The implant produced a new tendon in the defect area in which the diameter was comparable to the intact contralateral tendon while the regenerated tissue in the ICTs had significantly lower diameter than the treated and intact tendons.

The implants used in the present study were biodegradable and biocompatible *in vivo*. At least five possible biological responses have been suggested after implantation of extracellular matrices (ECM) including: 1) ECM non-incorporating responses (a) encapsulation (b) rejection and 2) ECM incorporating responses (a) resorption (b) integration with progressive degradation (c) adoption and adaptation [12], [57]. The results of the present study suggested that the implants were not rejected because no marked inflammatory reaction was present after the inflammatory stage of tendon healing and no giant cells were observed, while the remnants of the collagen and PDS scaffolds were present in the injured area. The biological response to this artificial tendon was a mixture of absorption, integration with progressive degradation, adoption and adaptation [57]. At fibroplasia and remodeling phases of tendon healing the remnants of the collagen implant were slowly absorbed and/or were infiltrated by the fibroblasts and were accepted as a part of the new tendon [58]. The immune response is a primary constituent of the host recognition to injury and implanted materials. Lymphocytes and helper T-cells (Th) in particular modulate the adaptive immune response through secreted cytokines [18]. Allman et al [17] showed subcutaneous implantation of small intestinal submucosa (SIS) in mice induces a Th2-associated immune response. This type of response is characterized by IL-4, IL-5, IL-6, and IL-10 cytokine production. It is anti-inflammatory in nature and accompanies xenograft acceptance. In contrast, the Th1 immune response typically induced through the IL-12 pathway is characterized by IL-2, IFN-g, and TNF-b expression. This type of immune response is inflammatory in nature and is associated with xenograft rejection [17]. Badylak et al [20] showed that after SIS implantation in dogs, an intense mononuclear cell response and infiltration into the implant was seen at 7, 14 and 28 DPI. These mononuclear cells were predominantly of an M2 phenotype at all-time points. By 16 weeks there was no evidence of the SIS and the surgical site was characterized by organized collagenous connective tissue, skeletal muscle tissue, and occasional M2 mononuclear cells. They have suggested that the M2 macrophage response is associated with an organized, site-appropriate tissue-remodeling outcome and absence of persistent inflammation. These studies may cover the results of the present study and explain why the collagen implant was not rejected.

Although the implants were cytocompatible *in vitro* and biocompatible *in vivo*, our records suggest that application of the collagen and collagen-PDS implants can affect the peripheral blood profile. In fact, our hematological results were in accordance with our histologic findings. While the neutrophil count was reduced in the peripheral blood profile, their number significantly increased in the injured area, especially in the inflammatory phase of tendon healing. However, this issue was not found to be a major concern because these changes in the peripheral blood were compensated by time and the animals were all clinically normal with no changes in their appetite or mental behavior. This issue may be clinically relevant to those patients who are going to be treated by these implants and have immune deficiency (e.g. HIV). Based on the results obtained from both the hematological and histopathological studies, although the number of lymphocytes in the peripheral blood of the treated animals was higher than the controls at 60 DPI, but regarding the histologic results the number of lymphocytes was lower in the treated tendons as compared to the controls. It seems at 60 DPI, the inflammation has subsided in the treated lesions compared to the controls. Greater lymphocytosis in the peripheral blood of the treated animals may be due to the drainage of the implant’s remnants into the regional lymph nodes. After implantation of the implants in the defect area, the inflammatory cells gradually degraded the implants and removed it from the defect area. The removed implant’s particles were possibly transferred into the regional lymph nodes by lymphatic vessels and resulted in proliferation of lymphocytes [59], [60]. Therefore, more lymphocytes in the peripheral blood of the treated animals, suggest that the regional lymph nodes may have been engaged with the degraded parts of the implants and are still active to completely lyse the scaffold remnants [59], [60]. This may explain why higher number of lymphocytes were needed by the treated animals.

Despite these explanations, our methodology had this limitation in which we did not compare the peripheral blood profile of each animal at different time points. We just obtained a sample from each animal at one time point (euthanasia) because repeated blood sampling could alter the healing potential of tendon and the reduced blood volume may have an impact on the clinical status of animals. In addition, we statistically compared the differences between a sufficient numbers of animals.

In such a large defect model in which more than 75% of the Achilles tendon was discarded, more time is needed for the treatment strategy to affect the quality of new tendon. The newly regenerated treated tendon, although having significantly more improved structural and functional performance compared to the control lesions, the differences between the treated tendons and the intact tendons were high, supporting the above statement in which the healing tendons need more time to be remodeled in order to increase their biomechanical performance. However, it has been suggested that despite intensive remodeling, the biomechanical characteristics of the intact tendon is never achieved even after many years [5], [12].

One of the merits of the bioimplants is that they are gradually degraded and can collaborate at different stages of tendon healing, so that after an optimum time (e.g. 60 DPI) most of the implants are replaced by the new tendon and the amount of foreign material in the healing tissue is extremely low. Nishimoto et al [61] tested the stent-shaped poly-L-lactic acid in a medial collateral ligament defect model in rabbits. They showed that despite the optimum biomechanical properties of the repaired area, the implant was not degraded and no new tissue replaced the scaffold after 16 weeks. In another study, Sato et al [11] investigated the effects of braided artificial tendons on experimentally induced tendon defect model in rabbits and showed that the biomechanical properties of different synthetic based artificial tendons (e.g. polylactic acid) reduced after implantation in the defect area. After 26 weeks, the implants were present and no tendinous tissue was replaced.

Another merit is that there is no need for administration of the immune suppressive drugs which are necessary for tissue transplantation, because the effectiveness of such bioimplant is completely dependent on the immune response so that the potential risk of infection and transmissible disease decreases. Such implants are cytocompatible, which means the stem cells can be grown in their architecture to produce a viable graft [62]. This characteristic together with the physical characteristics of the implants, led in assembling the bioimplants with healing promotive factors such as glycosaminoglycans and growth factors, and stem cells to improve their healing efficacy [12]. This could be an area of interest for those researchers aiming to improve tendon healing in near future.

The most important results necessary for clinical translation have been provided in this article, perhaps other molecular and biochemical aspects of the implants could be determined in future studies. Although the implants were cytocompatible *in vitro* and biocompatible in rabbits, it should be remembered that animal studies are an approximation, whereby each animal has its own advantages and disadvantages [62]. We suggest testing the biocompatibility behavior of the implant in humans in which there is a need to insert the implant subcutaneously and estimate the tolerance of the human body. If the human biocompatibility is confirmed, the bioimplants could then be designed for reconstructing many soft and hard connective tissues.

In conclusion, the collagen implant and PDS sheath were cytocompatible *in vitro* and biocompatible and biodegradable *in vivo*. They were effective in the healing of a large Achilles tendon defect model in rabbits and reduced the peritendinous adhesion, muscle fibrosis and atrophy, increased the collagen content and alignment of the new tendon and improved the biomechanical properties of the healing tendons. The implants, based on the reasons mentioned above, improved the function of the animals so that the treated animals had better weight bearing and physical activity at 60 DPI compared to the controls. The implants mostly activated the intrinsic mechanism of tendon healing and the collagen implant had the most crucial role in tendon healing, while addition of PDS sheath over the collagen implant had an ancillary role in tendon healing but reduced tendon adhesion and improved alignment of the new tendon more than those treated with collagen implant alone. The implants were not rejected by the host immune response but they were immunogenic and were gradually absorbed and their remnants collaborated in different stages of tendon healing. These remnants acted as scaffolds and aligned the new tendon and then were completely absorbed at later stages of tendon healing or were accepted as a part of the new tendon. Unlike the treated lesions, the unassisted tendon healing occurred in the control tendons failed to produce a new tendon and a loose areolar connective tissue was the sole regenerated tissue in the defect. Application of these bioimplants was found to be safe and could be suggested as an alternative of tissue grafts for large tissue defects when sufficient autograft tissue is not available.

## Supporting Information

Figure S1 **Preparation of the collagen implant (Part 1).** The collagen solution was placed in the syringe pump with the needle charged at 6 kV with respect to the base plate (A). The nanofibers were harvested and mixed with fresh collagen solution (B), incubated for final polymerization (C). This hybridized collagen gel was dried (D) and cut into rectangular strips to form several prostheses (E) which were cross-linked (F), sterilized and then dried (G). Each arrow shows a next step (A to G).
(TIF)

Figure S2 **Preparation of the collagen implant (Part 2).** (A) The electrospun collagen nanofibers are collected in the gap collector (Arrows). B: after the electrospinning, the door of the box closed and the collagen solution was added through the collagen valve both over and under the collector. (C) The collector has been removed and the collagen solution and the electrospun collagen fibers were combined together. (D) The box was placed at the 4°^C^ for 48 hours to produce large fibers. The polymerization of the larger collagen fibers is started close to the electrospun collagen sheet. (E) The polymerization is completed and the nano and micro collagen fibers are formed so that the hybridized collagen cube is seen. The electrospun collagen matrix acted as a scaffold for the newly polymerized collagen fibers and improved their alignment along its fiber orientation. An electromagnetic field was applied during the polymerization in order to improve the final alignment of the fibers.
(TIF)

Figure S3 ***In vitro*** **findings.** (A) SDS-PAGE images of type I collagen using 6% poly-acrylamide gel to examine the purification of collagen extracts. Lane a: molecular marker, lane b: bovine collagen. (B) Histologic section of the constructs after 20 days of cell culture. The proliferating fibroblasts are infiltrated to the implant and proliferated. (C–E) Cell viability was determined by live/dead cell assay using fluorescein diacetate (live) and propidium iodide (dead). (C, D and E) shows day 5, 10 and 20 after cell seeding. Almost all of the fibroblasts are green, indicating the cells are live. The lack of Propidium iodide stained dead cells (red) supports the idea that normal rat fibroblasts have attached to the scaffold and that the majority of the cells are viable. (F and G) Surface and inside of the collagen implant after 20 days of cell seeding, respectively. The cells proliferated (arrows) and produced matrix.
(TIF)

Table S1 **Clinical scoring criteria.**
(DOC)

Table S2 **Ultrasonographical scoring criteria.**
(DOC)

Table S3 **Gross morphological scoring criteria.**
(DOC)

Table S4 **Histological scoring criteria.**
(DOC)

Table S5 **Base scoring system used for defining the ultrastructure analysis (SEM).**
(DOC)

Protocol S1 **Preparation of the implants and quality control tests.**
(DOC)
