# Supplementary figures and images for: Tendon Tissue Engineering and Its Role on Healing of the Experimentally Induced Large Tendon Defect Model in Rabbits: A Comprehensive In Vivo Study
Source: PLoS One. 2013 Sep 5;8(9):e73016. doi: 10.1371/journal.pone.0073016 (PMC3764104; doi:10.1371/journal.pone.0073016)

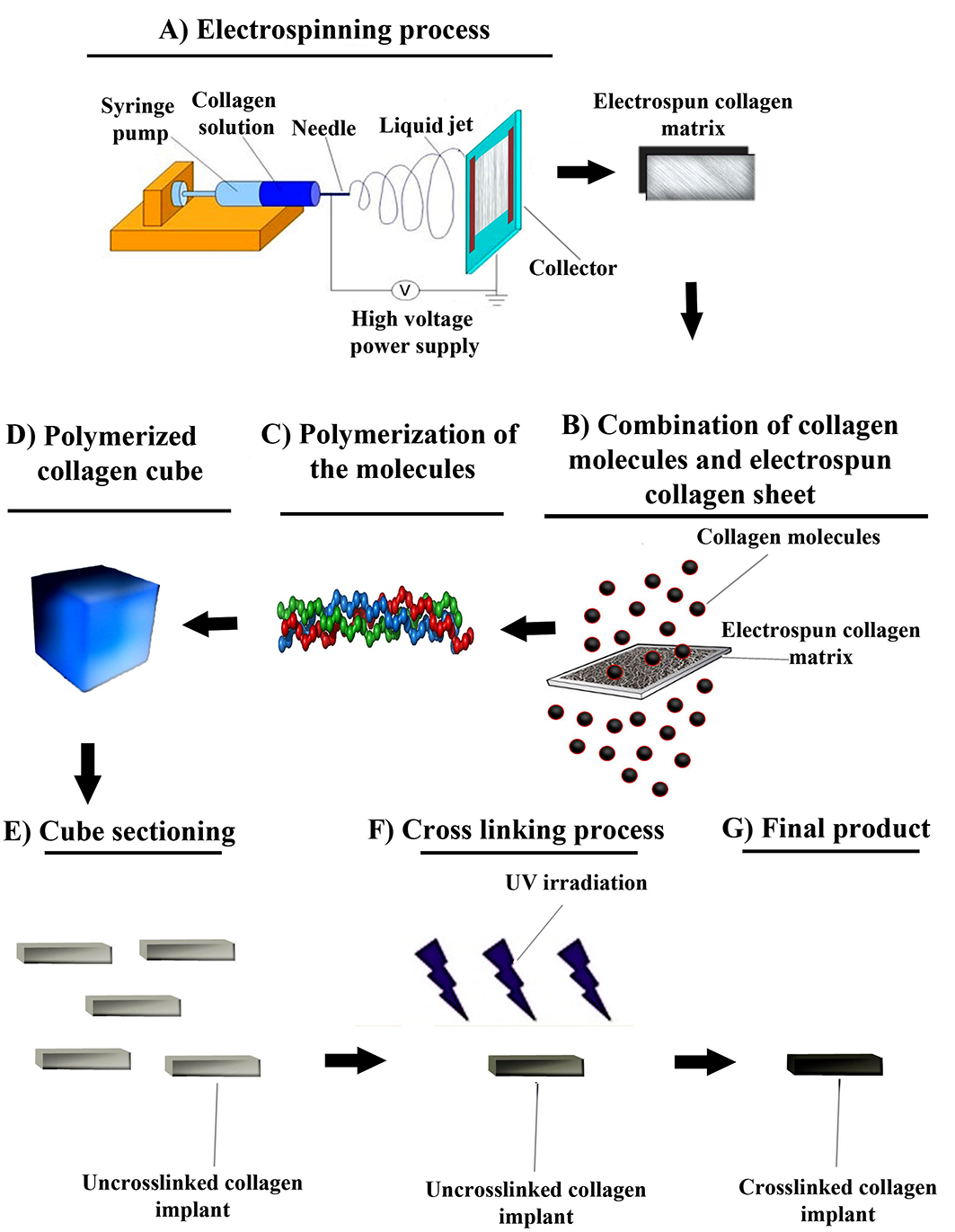

Supplement: Figure S1 — Preparation of the collagen implant (Part 1). The collagen solution was placed in the syringe pump with the needle charged at 6 kV with respect to the base plate (A). The nanofibers were harvested and mixed with fresh collagen solution (B), incubated for final polymerization (C). This hybridized collagen gel was dried (D) and cut into rectangular strips to form several prostheses (E) which were cross-linked (F), sterilized and then dried (G). Each arrow shows a next step (A to G). (TIF) [file pone.0073016.s001.tif]

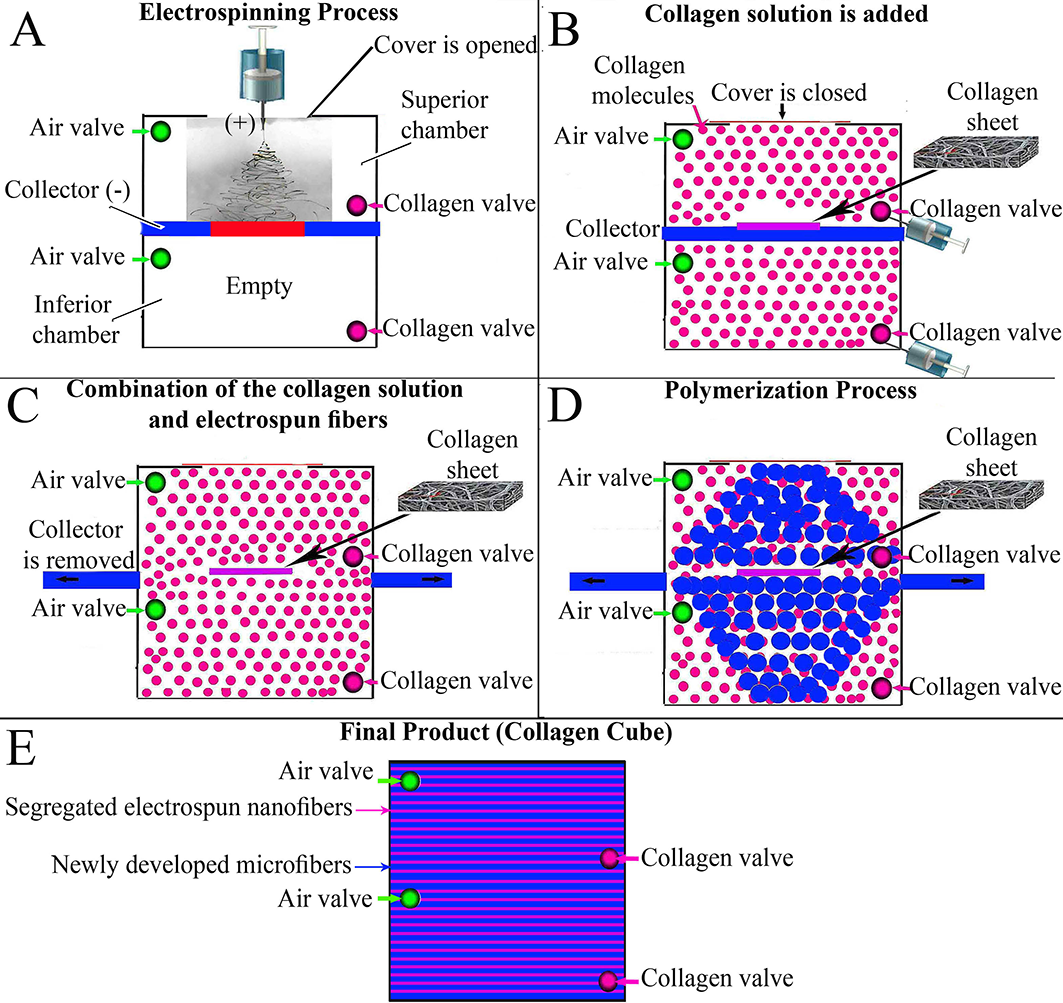

Supplement: Figure S2 — Preparation of the collagen implant (Part 2). (A) The electrospun collagen nanofibers are collected in the gap collector (Arrows). B: after the electrospinning, the door of the box closed and the collagen solution was added through the collagen valve both over and under the collector. (C) The collector has been removed and the collagen solution and the electrospun collagen fibers were combined together. (D) The box was placed at the 4°C for 48 hours to produce large fibers. The polymerization of the larger collagen fibers is started close to the electrospun collagen sheet. (E) The polymerization is completed and the nano and micro collagen fibers are formed so that the hybridized collagen cube is seen. The electrospun collagen matrix acted as a scaffold for the newly polymerized collagen fibers and improved their alignment along its fiber orientation. An electromagnetic field was applied during the polymerization in order to improve the final alignment of the fibers. (TIF) [file pone.0073016.s002.tif]

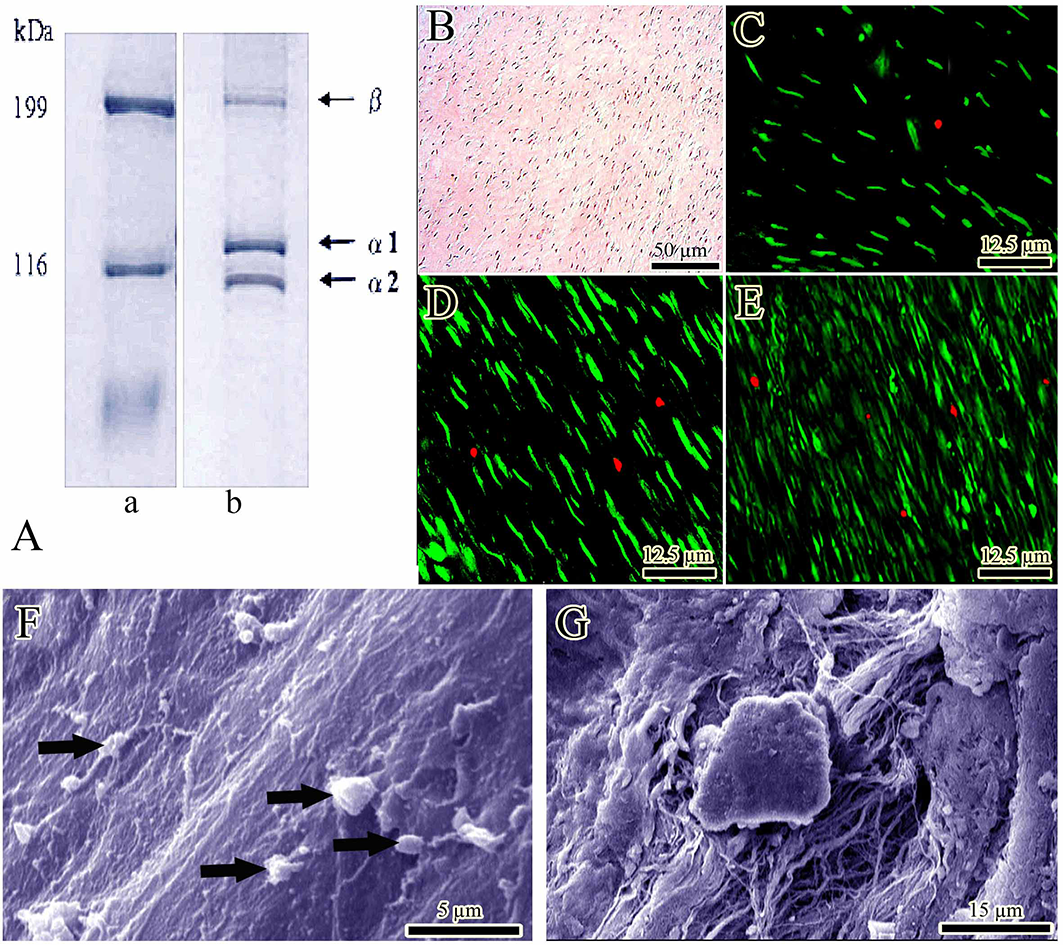

Supplement: Figure S3 — In vitro findings. (A) SDS-PAGE images of type I collagen using 6% poly-acrylamide gel to examine the purification of collagen extracts. Lane a: molecular marker, lane b: bovine collagen. (B) Histologic section of the constructs after 20 days of cell culture. The proliferating fibroblasts are infiltrated to the implant and proliferated. (C–E) Cell viability was determined by live/dead cell assay using fluorescein diacetate (live) and propidium iodide (dead). (C, D and E) shows day 5, 10 and 20 after cell seeding. Almost all of the fibroblasts are green, indicating the cells are live. The lack of Propidium iodide stained dead cells (red) supports the idea that normal rat fibroblasts have attached to the scaffold and that the majority of the cells are viable. (F and G) Surface and inside of the collagen implant after 20 days of cell seeding, respectively. The cells proliferated (arrows) and produced matrix. (TIF) [file pone.0073016.s003.tif]
