# Supplementary material for: Tendon Tissue Engineering and Its Role on Healing of the Experimentally Induced Large Tendon Defect Model in Rabbits: A Comprehensive In Vivo Study
Source: PLoS One. 2013 Sep 5;8(9):e73016. doi: 10.1371/journal.pone.0073016 (PMC3764104; doi:10.1371/journal.pone.0073016)
Supplement: Table S1 — Clinical scoring criteria. (DOC) [file pone.0073016.s004.doc]

**Table S1: Clinical scoring criteria**

| **The tarsal flexion degree of the injured leg compared to the normal leg, both in the cage and on the floor** | | | | | | | |
| --- | --- | --- | --- | --- | --- | --- | --- |
| **Score** | **Between legs** | | | | **Estimation of the degree** | | **Condition** |
| **0** | - Equal | | | | - 75-90 | | Normal |
| **1** | - Non equal | | | | - 50-74 | | Mild |
| **2** | - Non equal | | | | - 30-50 | | Moderate |
| **3** | - Non equal | | | | - >30 | | Severe |
| **4** | - Non equal | | | | - <15 | | Extensively severe |
| **Weight distribution of each animal on the hind limbs, in the cage and on the floor** | | | | | | | |
|  | **Weight distribution between limbs** | **Weight distribution between hind legs** | | **The most weight bearing legs** | | **The injured left hind leg condition** |  |
| **0** | - Mostly hind limb | - Equal | | - Both hind limbs | | - Weight bearing | Normal |
| **1** | - Mostly hind limb | - Not equal | | - Right hind limb | | - Weight bearing | Mild |
| **2** | - Mostly forelimbs | - Not equal | | - Both forelimbs & right normal hind limb | | - Weight bearing | Moderate |
| **3** | - Not Equal | - Not equal | | - Both forelimbs and right normal hind limb | | - Non weight bearing | Severe |
| **4** | - Not Equal | - Not equal | | - Non weight bearing (sternal recumbency) | | - Non weight bearing | Extremely severe |
| **Pain in palpation of the injured area and pain in complete extension of the injured leg** | | | | | | | |
| **0** | - No reaction | | | | | | Normal |
| **1** | - Occasional vocalization | | | | | | Mild |
| **2** | - Frequent vocalization | | | | | | Moderate |
| **3** | - Vociferous vocalization, withdraw limb, bites, struggles | | | | | | Severe |
| **Heel and toe position of the injured leg (left hind paw)** | | | | | | | |
|  | **Heel** | | **toe** | | | |  |
| **0** | - Up | | - Down | | | | Normal |
| **1** | - Near the floor (up) | | - Down | | | | Mild |
| **2** | - Down | | - Down | | | | Moderate |
| **3** | - Down | | - Up | | | | Severe |
| **Swelling at the injured area (left hind paw)** | | | | | | | |
| **0** | Is not tender, warm and bowed | | | | | | Normal |
| **1** | Slightly warm and bowed, color is not changed | | | | | | Mild |
| **2** | Tenderness, bowed and completely warm. Color in not changed | | | | | | Moderate |
| **3** | Obvious tenderness, bowed and warm. Color changed. | | | | | | Severe |
