# Supplementary material for: Tendon Tissue Engineering and Its Role on Healing of the Experimentally Induced Large Tendon Defect Model in Rabbits: A Comprehensive In Vivo Study
Source: PLoS One. 2013 Sep 5;8(9):e73016. doi: 10.1371/journal.pone.0073016 (PMC3764104; doi:10.1371/journal.pone.0073016)
Supplement: Table S2 — Ultrasonographical scoring criteria. (DOC) [file pone.0073016.s005.doc]

**Table S2: Ultrasonographical scoring criteria**

| **Score** | 1. **Echogenicity** | 1. **Hyper echogenic area / hypo echogenic area of the tendons (homogeneity)** | 1. **Transverse movement of the tendon (Index for peritendinous adhesion)** |
| --- | --- | --- | --- |
| **0** | - Normal echogenicity | - Smooth (homogenous) echogenicity | - Free to move |
| **1** | - Slightly hyper-echoic | - Non-smooth (heterogeneous) echogenicity (mild) | - Movable in one direction (left or right) |
| **2** | - Hyper-echoic | - Non-smooth echogenicity (moderate) | - Movable in one direction with force (left or right) |
| **3** | - Hypo-echoic | - Amputated view or non-smooth echogenicity (severe) | - Fixed or non-movable |
|  | 1. **Diameter of the injured tendon / intact contralateral (regeneration volume)** | 1. **Diameter of the peritendinous low echogenic area / diameter of the high echogenic tendon (intensity of the peritendinous adhesion)** | 1. **Diameter of the proximal part of the tendon / diameter of the distal part of the tendon (regenerative proportion)** |
| **0** | - 100% | - Less than 10% | - 110-140% |
| **1** | - 90% | - 11-25% | - 91-109% |
| **2** | - 101-110% | - 26-50% | - 75-90% |
| **3** | - 75%-100% | - 50-100% | - 50-74% |
| **4** | - 50%-74% | - 100-150% | - 25-49% |
| **5** | - 0-50% | - 151% and more | - Less than 25% |
