# Supplementary material for: Tendon Tissue Engineering and Its Role on Healing of the Experimentally Induced Large Tendon Defect Model in Rabbits: A Comprehensive In Vivo Study
Source: PLoS One. 2013 Sep 5;8(9):e73016. doi: 10.1371/journal.pone.0073016 (PMC3764104; doi:10.1371/journal.pone.0073016)
Supplement: Table S3 — Gross morphological scoring criteria. (DOC) [file pone.0073016.s006.doc]

Table S3: Gross morphological scoring criteria

| **Gross pathological findings (visual observation)** | | | |
| --- | --- | --- | --- |
| **Score** | 1. **Peritendinous adhesion** | 1. **Hyperemia** | **Status** |
| **0** | - No adhesion | - No hyperemia, shiny glistening surface appearance | Normal |
| **1** | - Tendon was easily detached from the surrounding tissues by blunt dissection | - Only in the paratenon | Mild |
| **2** | - For detachment from the surrounding tissues, tendon needed little sharp dissection | - It was extended to the tendon proper but it was not severe in nature | Moderate |
| **3** | - For detachment from the surrounding tissues, tendon needed completely sharp dissection | - It was extensively extended to the tendon proper and made its appearance more pink and dark | Severe |
| **Score** | 1. **General appearance** | **4) Muscle Atrophy** | **Status** |
| **0** | - Tendon is a unit structure (the tendinous tissue is organized as a separate tissue and could be differentiated from the surrounding structure) and is continued between the gastrocnemius muscle and calcaneal tuberosity with the same diameter and homogeneity | - The transvers diameter of the largest bulk of the muscle is more than or equal to 350% of the transvers diameter of the largest part of the Achilles tendon at its mid part. | Normal |
| **1** | - Same as above but the diameter of the injured area is larger than the proximal and distal parts of the tendon. The tendon generally is a unit structure. | - The transvers diameter of the largest bulk of the muscle is more than or equal to 250% of the transvers diameter of the largest part of the Achilles tendon at its mid part. | Mild |
| **2** | - Same as above but the diameter of the injured area is lower than the proximal and distal parts of the tendon. The tendon generally is a unit structure. | - The transvers diameter of the largest bulk of the muscle is more than or equal to 200% of the transvers diameter of the largest part of the Achilles tendon at its mid part. | Fairly moderate |
| **3** | - The injured area of the tendon is not a unit structure but the proximal and the distal parts of the tendon are a unit structure. | - The transvers diameter of the largest bulk of the muscle is more than or equal to 150% of the transvers diameter of the largest part of the Achilles tendon at its mid part. | Moderate |
| **4** | - The whole of the tendon is not a unit structure. No structure similar to tendon is seen between the gastrocnemius muscle and calcaneal tuberosity and the posterior tibialis tendon is seen. In normal condition Achilles tendon covers the posterior tibialis muscle but in this condition due to the lysis of the Achilles tendon the posterior tibialis muscle could be seen. | - The transvers diameter of the largest bulk of the muscle is more than or equal to 10% of the transvers diameter of the largest part of the Achilles tendon at its mid part. | Severe |
| **Score** | **5) Muscle fibrosis** | | **Status** |
| **0** | - No fibrosis is seen in the gastrocnemius muscle and the tendinous portion of the Achilles is the only connective tissue that covered the muscle | | Normal |
| **1** | - Mild fibrosis is seen in the muscle but more than 75% of the muscle has red color and had gross appearance similar to the muscle | | Mild |
| **2** | - Between 50 to 74% of the muscle has the characteristics of the muscle but the fibrosis is significant | | Moderate |
| **3** | - More than 50% of the muscle shows fibrosis and the fibrous tissue filled the spaces between muscle fibers. | | Severe |
| **4** | - No muscular characteristic could be seen in the gastrocnemius muscle because all of the muscle was substituted by fibrous tissue | | Extremely severe |
