# Supplementary material for: Tendon Tissue Engineering and Its Role on Healing of the Experimentally Induced Large Tendon Defect Model in Rabbits: A Comprehensive In Vivo Study
Source: PLoS One. 2013 Sep 5;8(9):e73016. doi: 10.1371/journal.pone.0073016 (PMC3764104; doi:10.1371/journal.pone.0073016)
Supplement: Table S4 — Histological scoring criteria. (DOC) [file pone.0073016.s007.doc]

Table S4: Histological scoring criteria

| **Histopathologic analysis** | | | |
| --- | --- | --- | --- |
| **Score** | **1) Alignment** | **2) Perivascular edema** | **Status** |
| **0** | - Collagen fibers were longitudinally oriented in only one direction and the tenoblasts and tenocytes were laid longitudinally along their orientation | - No edema | Normal |
| **1** | - Collagen fibers were longitudinally oriented in one direction pattern but there were few areas of unorganized collagen fibers in the field | - Presence of edema just around small vessels | Mild |
| **2** | - Collagen fibers were not longitudinally oriented and the irregular orientation pattern was predominant | - Presence of edema around small and medium sized vessels | Moderate |
| **3** | - There was no obvious pattern and the collagen fibers were disorganized | - Presence of edema around all types of vessels | Severe |
| **Score** | **3) Tissue Maturity** | | **Status** |
|  | **A) the appearance of the collagen fibers** | **B) cellular populations** |  |
| **0** | - More than 75% collagen fibers are dense and they have large size | - More than 75% are fibrocytes | Normal or near normal |
| **1** | - More than 50% of the collagen fibers are dense and they are of large size | - More than 50% are fibrocytes | Highly mature |
| **2** | - More than 25% of the collagen fibers are dense and they are medium sized | - More than 25% are fibrocytes | Moderately mature |
| **3** | - The collagen fibers are not dense but they are medium sized | - More than 75% are fibroblasts | Immature |
| **4** | - The collagen fibers are not dense and they are of small sized | - The inflammatory cells are predominant | Highly immature |
| **Score** | **4) Crimp pattern** | **5) Vascularity (at remodeling stage)** | **Status** |
| **0** | - More than 75% of the collagen fibers in the light microscopic field are wavy | - No vascular structures are visible in the tissue sections. | Normal |
| **1** | - 50%-75% of the collagen fibers in the light microscopic field are wavy | - Less than 10% of the tissue density belongs to vessels. | Optimum (remodeled) |
| **2** | - 25%-50% of the collagen fibers in the light microscopic field are wavy | - Less than 25% of the tissue density belongs to vessels | Early remodeling  (fairly acceptable) |
| **3** | - Less than 25% of the collagen fibers in the light microscopic field are wavy | - Less than 50% of the tissue density belongs to vessels | Fibroplasia (bad) |
| **4** | - No crimp pattern is seen | - Less than 75% of the tissue density belongs to vessels | Early fibroplasia or degenerative changes (extremely bad) |
| **5** |  | - More than 75% of the tissue density belongs to vessels | Healing is not in progress and the newly regenerated tissue is only vascularized. |
