# Supplementary material for: Tendon Tissue Engineering and Its Role on Healing of the Experimentally Induced Large Tendon Defect Model in Rabbits: A Comprehensive In Vivo Study
Source: PLoS One. 2013 Sep 5;8(9):e73016. doi: 10.1371/journal.pone.0073016 (PMC3764104; doi:10.1371/journal.pone.0073016)
Supplement: Table S5 — Base scoring system used for defining the ultrastructure analysis (SEM). (DOC) [file pone.0073016.s008.doc]

**Table S5: Base scoring system used for defining the ultrastructure analysis (SEM)**

| **1) Alignment** | | | | | |
| --- | --- | --- | --- | --- | --- |
| **Score** | **Status** | | **Directions of the collagen fibrils** | | **Direction of the fibroblasts and fibrocytes** |
| **0** | Near normal | | - Most of them are aligned in one directions | | - Most of them are laid in a direction of collagen fibrils |
| **1** | Highly aligned | | - More than ¾ of the collagen fibrils are aligned in one directions | | - More than ¾ of the cells are laid in a direction of collagen fibrils |
| **2** | Moderately aligned | | - More than ½ of the collagen fibrils are aligned in one directions | | - More than ½ of the cells are laid in a direction of collagen fibrils |
| **3** | Fairly aligned | | - More than ¼ of the collagen fibrils are aligned in one directions | | - More than ¼ of the cells are laid in a direction of collagen fibrils |
| **4** | Amorphous | | - Collagen fibrils are not aligned in one direction. | | - Most of the cells are not laid in the direction of the collagen fibrils |
|  | **2) Maturity of the collagen fibrils** | | | | |
| **Score** | **Status** | **Description** | | | |
| **0** | Normal | - Collagen fibrils are distributed in multimodal pattern. Minimum of five different category of fibril’s diameter (extremely small (0-64nm), Small (65-102nm), medium (103-153 nm), large (154-256nm), extremely large (257-307nm)) is seen at ultra-micrographs. | | | |
| **1** | Highly matured | - Collagen fibrils are distributed in multimodal pattern. Four different category of fibril’s diameter (extremely small (0-64nm), Small (65-102nm), medium (103-153nm), large (154-256nm)) is seen at ultra-micrographs. | | | |
| **2** | Matured | - Collagen fibrils are distributed in multimodal pattern. Three different category of fibril’s diameter (extremely small (0-64nm), Small (65-102), medium (103-153nm)) is seen at ultra-micrographs. | | | |
| **3** | Immature | - Collagen fibrils are distributed in bimodal pattern. Two different category of fibril’s diameter (extremely small (0-64nm), Small (65-102nm)) is seen at ultra-micrographs. | | | |
| **4** | Highly immature | - Collagen fibrils are distributed in unimodal pattern. Only one category of fibril’s diameter (extremely small (0-64nm) is seen at ultra-micrographs. | | | |
| **Score** | **3) Crimp pattern** | | |  |  |
| **0** | - More than 75% of the collagen fibers in the field are wavy | | |  |  |
| **1** | - 50%-75% of the collagen fibers in the field are wavy | | |  |  |
| **2** | - 25%-50% of the collagen fibers in the field are wavy | | |  |  |
| **3** | - Less than 25% of the collagen fibers in the field are wavy | | |  |  |
| **4** | - No crimp pattern is seen | | |  |  |
