# Supplementary material for: Tendon Tissue Engineering and Its Role on Healing of the Experimentally Induced Large Tendon Defect Model in Rabbits: A Comprehensive In Vivo Study
Source: PLoS One. 2013 Sep 5;8(9):e73016. doi: 10.1371/journal.pone.0073016 (PMC3764104; doi:10.1371/journal.pone.0073016)
Supplement: Protocol S1 — Preparation of the implants and quality control tests. (DOC) [file pone.0073016.s009.doc]

**Tendon tissue engineering and its role on healing of the experimentally induced large tendon defect model in rabbits: a comprehensive *in vivo* study**

**Abdolhamid Meimandi-Parizi, Ahmad Oryan and Ali Moshiri**

**Protocol S1**

1. **Preparation of the collagen implant**
   1. **Collagen electrospinning**

Collagen type I was extracted from the bovine superficial digital flexor tendon according to the methods of Foltran et al [1]. The purity of the type I collagen was assessed by SDS/PAGE using 6% separating gels. The gel was then stained overnight with 0.017% (w/v) Coomassie blue R-250 (Bio-Rad®, Hercules, USA) in 38.8% methanol and 6.8% acetic acid. Subsequently, the gel was destained with 5% methanol and 5% acetic acid for 48 hours.

The electrospinning solution was prepared by mixing collagen (type I acid soluble from bovine tendon) in acetic acid (>99%; EMD, San Diego, CA). The solution (7.5 % (w/w) collagen) was loaded into a syringe (Air-tite Products, Virginia Beach, VA) with a blunt-ended needle (18, gauge). The syringe was then placed on a syringe pump (New Era Pump Systems, Inc., NY) and directed toward a grounded collection device. Voltages of 6 kV were applied to the needle when the flow rate on the syringe pump was set to dispense at 0.15 mL/h. Uniformly aligned fibers were constructed by electrospinning onto a dual plate device. Dual plate devices used in this experiment consisted of two 2.5×0.5 cm copper strips attached to a gap substrate with Gluseal (Glustitch, Gulf Road Point Roberts, WA). The gap substrate was quartz glass (McMaster-Carr, Elmhurst, IL) with an electrical resistivity of 10^20^ Ωm. The gap between the copper strips was 1 cm, and each copper strip had a separate grounding wire. The dual plates were 4 cm away from the needle tip. The copper plates were removed after electrospinning [2]. Highly aligned and large electrospun collagen fibers with a diameter of 272 ± 183 nm were produced successfully (Fig. 1B, S1 and S2 A). The collagen matrix was then let to be dried at room temperature overnight to remove remaining solvents.

- 1. **Production of tridimensional collagen implant**

After electrospinning, acid-solubilized bovine tendon type I collagen was mixed with phosphate buffered saline (PBS) (Sigma, 10 ×) and NaOH (0.1N) to prepare a collagen solution with pH between 7.2 and 7.4 and concentration of 10 mg/ml. This mixture was immediately pipetted into a syringe and injected into a custom made chamber (10 × 10 × 10 cm) box containing electrospun collagen matrix. The box was placed in an incubator at 4^o^C for 48 hours to polymerize the collagen gel and to produce large fibers. The collagens were aligned under 12 Tesla magnetic fields (CRETA, Grenoble) during polymerization [3]. The range of the ﬁber diameters was 1.82 µm to 3.19 µm. The electrospun collagen matrix (2D) acted as a core with its fibro-conductive characteristics and improved alignment of the newly formed collagen fibers. The collagen composite was cut into several pieces of the same size and shape as the rabbit's Achilles apparatus (L=2cm, H=3. 5 mm, W=3 mm) (Fig. S1 and S2).

- 1. **Cross linking of the collagen implant**

For crosslinking, the implants were suspended in iso-osmolar 0.1% riboflavin solution with Dextrane T500 (as a photosensitizer), 20 minutes before the irradiation to allow sufficient saturation of the implant. The implants were irradiated for 60 minutes with UV (wavelength of 365 nm) at a working distance of 5 cm, with an irradiance of 3 mW/cm^2^ corresponding to a surface dose of 5.4 J/cm^2^, (UV-X^TM^, Peschke Meditrade, Cham, Switzerland). During irradiation, drops of riboflavin solution were applied to the implant every 4 to 5 minutes to sustain the necessary concentration of the riboflavin. The implants were neutralized with distilled water four times (30 min each) (Fig. S1) [4].

- 1. **Preparation of polydioxanone artificial sheath**

To produce the bio-synthetic implant, polydioxanone nano scaled plates were purchased (PDS plate, Ethicon, Johnson & Johnson, USA), sectioned at 300 nm, melted and wrapped around each collagen piece to cover the implant at the periphery (Fig. 1 H-L).

- 1. **Maintenance of the implants and sterility**

The final product was repeatedly washed with distilled water to remove the residual enzymes and chemical reagents, let to be dried overnight, received 100 Gray g-radiation for 120 min and then suspended in ethanol 96% to produce and maintain its sterility until surgery. Two hours before surgery, the implant was dried to evaporate the ethanol residues and repeatedly washed with distilled water (10 min×12 times) and finally suspended in isotonic saline solution to soften the implant for better surgical handling [5].

1. **Initial tests of the scaffold**
   1. **Morphology of the scaffolds**

Morphology of the scaffolds was studied by SEM (*n=10*). Porosity (free spaces between the collagen fibers) was deﬁned as the ratio of empty area to the total area [2] (Fig. 1).

- 1. **Microbiological tests**

The scaffolds were tested for sterility immediately after sterilization so that they were immersed in a Nutrient Agar Broth (NEOGEN Co. Lansing, MI, USA) to cultivate fastidious micro-organisms and maintained under agitation at 25 °C for 48 h. Non-sterile scaffolds (*n=10*) were used as negative control while the Graft jacket (Regenerative Tissue Matrix, Wright Medical Technology, Inc. San Antonio, TX, USA) was used as the positive control (*n=10*). Bioburden Challenge Test was also performed in a following manner: Test organisms that included Methicillin-resistant *Staphylococcus* *aureus* (MRSA; DMST 20645 lot no. 3273, NIH) and *Bacillus* *subtilis* (ATCC 6633 DMST 15896 lot no. 3479, NIH) were suspended in tryptic soy broth (TSB) (Sigma-Aldrich Co. LLC) to provide a final concentration of 10^2^ and 10^4^ cfu/mL. Inoculation of the test carrier was performed by using a micropipette to place 20 μL of the test suspension on the surface of scaffolds and left to dry in the incubator for 18 h at 37°C. The scaffolds were then sterilized as described before. After sterilization, the scaffolds were then transferred to test tubes containing TSB and were incubated at 25 °C for 7 days. The turbidity of the TSB was measured every day using a UV spectrophotometer at 625 nm wavelength and using McFarland Standards as a reference to estimate the number of colonies [6].

- 1. **Determination of endotoxin content**

The Limulus Amebocyte Lysate (LAL) (ToxinSensor^TM^ Gel Clot Endotoxin Assay Kit, GenScript Inc. Piscataway, NJ, USA) test was used according to the manufacturer's recommendation. The LAL reagent was mixed with the samples into the endotoxin-free vials, and then incubated at 37 °C for 60 minutes. Each vial was then inverted and checked whether a gel was formed.

- 1. **Cell seeding and cell culture**

Rat skin ﬁbroblasts (cell line CRL-1213) were obtained from the American Type Culture Collection (Manassas, VA) and cultured (37 ^o^C, CO_2_ 5%, pH=7.4) in Dulbecco’s Modiﬁed Eagle Medium supplemented with 10% fetal bovine serum, 20 U/ml penicillin, and 20 µg/ml streptomycin (Invitrogen, Carlsbad, CA). Cell culture medium was replaced every three days. Cells were passaged at conﬂuence and the 4–8^th^ passage ﬁbroblasts were used for the seeding. The sterile scaffolds were transferred into the wells of a custom-made 6-well culture chamber and seeded each with 1ml of 5.0 × 10^5^ cells.

The scaffolds were grown *in vitro* in a 5% CO_2_ incubator at 37 °C for 5, 10 and 20 days, with the medium being replaced every 3 days. The tissue constructs were removed from cell culture, and after routine histologic preparation were stained with hematoxylin and eosin and examined by a light microscope (Olympus, Tokyo, Japan) for regular cell counting and studying their distribution in the histopathologic fields. Number of cells per microscopic field in five fields per specimen (*n=10*) were counted [7,8].

- 1. **Cytocompatibility of the scaffolds**

Cell viability was determined by live/dead cell assay using ﬂuorescein diacetate (FDA, Molecular Probes, Invitrogen Corporation) (live) and propidium iodide (Cayman Chemical Company, Michigan, USA) (dead). The scaﬀolds with the ﬂuorescence stained cells were viewed under a Nikon ﬂuorescent microscope. A number of the live stained cells and dead stained cells were counted by the computer software (Image J, NIH, USA). The viability index was analyzed according to the following equation: Viability index= (number of viable cells/total number of cells) × 100 [8].

- 1. **Biomechanical testing of the scaffolds**

The un-cross-linked collagen implants, cross linked collagen implants and the hybridized collagen-PDS implants were subjected to the biomechanical test using the tensile testing machine (INSTRON® Tensile Testing Machine, London, UK). Scaffolds were hydrated for 24 h in PBS (pH 7.2) and drawn at a speed of 10 mm/min. The maximum load, maximum stress, strain and modulus of elasticity of the implants were extracted from the generated force-displacement and stress-strain curves [2].

- 1. **Determination of water binding capacity of the scaffolds**

Scaffold samples of about 5 mg dry weight were incubated in 3 ml PBS (pH 7.2) at 20^o^C. After 1 h, the wet weight was determined and the water-binding capacity calculated according to the following equation: water-binding capacity = (wet weight-dry weight) / (dry weight) [2,9].

1. ***In vitro* results**
   1. **Collagen purity**

Collagen extract contained two main α- chains (α_1_ and α_2_) and one β chain with very little contamination of other proteins. This suggested that collagen type I extract were pure with very little protein degradation product or other protein contamination (Fig. S3 A).

- 1. **Morphology of the scaffolds**

The transverse diameter of the electrospun fibers in 2D collagen matrix was 272 ± 183 nm (ranging 89 to 455 nm) (Fig. 1 B). Also the transverse diameter of the polymerized microfibers in the collagen gel (3D) was 2.54 ± 0.68 µm (ranging 1.82 to 3.19µm) (Fig. 1 C and D). In cross sections of the collagen implant, the porosity was 17.62 ± 4.88% (Fig. 1 E and F). The average diameter of these pores was 8.43 ± 4.27 µm (*n=100* pores). The proportion of the aligned collagen fibers/randomly formed collagen fibers were 71.27 ± 12.81/28.91 ± 4.22 (Fig. 1 C).

- 1. **Sterility of the scaffolds**

After 48 h incubation, the untreated controls (unsterilized scaffolds) produced signs of growth during sterility test whereas the Graftjacket and the sterilized scaffolds remained free of growth throughout the same time period and showed successful sterilization throughout the treatment durations. The result of bioburden test also showed that the sterilization technique was effective because the broth media was clear after challenging with high bacterial suspension even after 7 days incubation.

- 1. **Endotoxin level of the scaffolds**

The endotoxin levels of the collagen implants were below 0.25 EU/ml and no gel or turbidity was formed.

- 1. **Cell viability and cytocompatibility of the scaffolds**

Almost all of the fibroblasts were green, indicating the cells were live. Lack of propidium iodide (red) stained dead cells supports the idea that normal rat ﬁbroblasts were attached to the scaffold and that the majority of the cells were viable. A number of the proliferated cells in the scaffolds were 89.32 ± 12.84 (day 5), 202.17 ± 31.09 (day 10), and 358.92 ± 66.82 (day 20), at histology level (× 200) (Fig. S3B). The number of FDA stained viable cells in the scaffold (×200) was 84.71 ± 9.41 (day 5), 186.33 ± 27.91 (day 10), and 321.34 ± 49.23 (day 20) and was 94.83% (day 5), 92.16 % (day 10) and 89.52% (day 20) of the total cellularity (vitality index). The pattern of cell proliferation was homogenous in the scaffold (Fig. S3 C-E). The SEM images indicated that the fibroblasts well proliferated both on the surface and internal architecture of the implants and produced matrix (Fig. S3 F and G).

- 1. **Biomechanical characteristics of the scaffolds**

The maximum load ^N^ (95.37 ± 4.71^collagen-PDS^ vs. 28.33 ± 2.19 ^cross-linked collagen^ *vs*. 4.56 ± 1.92^un cross-linked collagen^, *P=0.001* for all), maximum stress ^N/mm2^ (9.08 ± 1.79 ^collagen-PDS^ vs. 2.69 ± 0.47 ^cross-linked collagen^ *vs*. 0.40 ± 0.11^un cross-linked collagen^, *P=0.001* for all), and modulus of elasticity ^kPa^ (610.46 ± 56.92 ^collagen-PDS^ *vs*. 43.81 ± 4.19 ^cross-linked collagen^ *vs*. 4.05 ± 1.28 ^uncross-linked collagen^) of the hybridized collagen-PDS implant (*n=10*) were significantly higher than those of the cross-linked (*n=10*) and un-cross-linked (*n=10*) collagen implants. Also the maximum strain% of the hybridized collagen-PDS implant (n=10) was significantly lower than those of the un-cross-linked (*n=10*) and cross-linked (*n=10*) collagen implants (14.81 ± 1.28 ^collagen-PDS^ *vs*. 61.34 ± 4.71 ^cross-linked collagen^ *vs*. 98.55 ± 7.23 ^uncross-linked collagen^, *P=0.001* for all).

- 1. **Water binding capacity of the scaffolds**

Water binding capacity of the un-cross-linked collagen scaffold (*n=10*), cross-linked collagen scaffold (*n=10*), collagen-PDS scaffold (*n=10*) and intact Achilles tendons of the rabbits (*n=10*) were 20.8 ± 1.8, 7.2±1.2, 1.8 ± 0.4 and 0.9 ± 0.2, respectively.

**References**

1. Foltran I, Foresti E, Parma B, Sabatino P, Roveri N. (2008) Novel biologically inspired collagen nanofibers reconstituted by electrospinning method. Macromol Symp 269: 111–118.
2. [Wray LS](http://www.ncbi.nlm.nih.gov/pubmed?term=Wray LS%5BAuthor%5D&cauthor=true&cauthor_uid=19193140), [Orwin EJ](http://www.ncbi.nlm.nih.gov/pubmed?term=Orwin EJ%5BAuthor%5D&cauthor=true&cauthor_uid=19193140). (2009) Recreating the microenvironment of the native cornea for tissue engineering applications. [Tissue Eng Part A](http://www.ncbi.nlm.nih.gov/pubmed?term=Recreating the Microenvironment of the Native Cornea for Tissue Engineering Applications)  15: 1463-1472.
3. [Dubey N](http://www.ncbi.nlm.nih.gov/pubmed?term=Dubey N%5BAuthor%5D&cauthor=true&cauthor_uid=11352087), [Letourneau PC](http://www.ncbi.nlm.nih.gov/pubmed?term=Letourneau PC%5BAuthor%5D&cauthor=true&cauthor_uid=11352087), [Tranquillo RT](http://www.ncbi.nlm.nih.gov/pubmed?term=Tranquillo RT%5BAuthor%5D&cauthor=true&cauthor_uid=11352087). (2001) Neuronal contact guidance in magnetically aligned fibrin gels: effect of variation in gel mechano-structural properties. [Biomaterials](http://www.ncbi.nlm.nih.gov/pubmed?term=Neuronal contact guidance in magnetically aligned) 22: 1065-1075.
4. [McCall](http://www.iovs.org/search?author1=A.+Scott+McCall&sortspec=date&submit=Submit) AS, [Kraft](http://www.iovs.org/search?author1=Stefan+Kraft&sortspec=date&submit=Submit) S, [Edelhauser](http://www.iovs.org/search?author1=Henry+F.+Edelhauser&sortspec=date&submit=Submit) HF, [Kidder GW](http://www.ncbi.nlm.nih.gov/pubmed?term=Kidder GW%5BAuthor%5D&cauthor=true&cauthor_uid=19643975), [Lundquist RR](http://www.ncbi.nlm.nih.gov/pubmed?term=Lundquist RR%5BAuthor%5D&cauthor=true&cauthor_uid=19643975), et al. (2010) Mechanisms of corneal tissue cross-linking in response to treatment with topical riboflavin and long-wavelength ultraviolet radiation (UVA). Invest Ophthalmol Vis Sci 51: 129-138.
5. [Stillaert FB](http://www.ncbi.nlm.nih.gov/pubmed?term=Stillaert FB%5BAuthor%5D&cauthor=true&cauthor_uid=18635258), [Di Bartolo C](http://www.ncbi.nlm.nih.gov/pubmed?term=Di Bartolo C%5BAuthor%5D&cauthor=true&cauthor_uid=18635258), [Hunt JA](http://www.ncbi.nlm.nih.gov/pubmed?term=Hunt JA%5BAuthor%5D&cauthor=true&cauthor_uid=18635258), [Rhodes NP](http://www.ncbi.nlm.nih.gov/pubmed?term=Rhodes NP%5BAuthor%5D&cauthor=true&cauthor_uid=18635258), [Tognana E](http://www.ncbi.nlm.nih.gov/pubmed?term=Tognana E%5BAuthor%5D&cauthor=true&cauthor_uid=18635258), et al. (2008) Human clinical experience with adipose precursor cells seeded on hyaluronic acid-based spongy scaffolds. Biomaterials 29: 3953e3959.
6. [Siritientong T](http://www.ncbi.nlm.nih.gov/pubmed?term=Siritientong T%5BAuthor%5D&cauthor=true&cauthor_uid=21671201), [Srichana T](http://www.ncbi.nlm.nih.gov/pubmed?term=Srichana T%5BAuthor%5D&cauthor=true&cauthor_uid=21671201), [Aramwit P](http://www.ncbi.nlm.nih.gov/pubmed?term=Aramwit P%5BAuthor%5D&cauthor=true&cauthor_uid=21671201). (2011) The effect of sterilization methods on the physical properties of silk sericin scaffolds. [AAPS Pharm Sci Tech](http://www.ncbi.nlm.nih.gov/pubmed?term=The Effect of Sterilization Methods on the Physical Properties of Silk Sericin Scaffolds) 12: 771-781.
7. Khanam N, Mikoryak C, Draper RK, Balkus KJ Jr. (2007) Electrospun linear polyethyleneimine scaffolds for cell growth. Acta Biomater 3: 1050–1059.
8. Zhao L, Burguera EF, Xu HH, Amin N, Ryou H, et al. (2010) Fatigue and human umbilical cord stem cell seeding characteristics of calcium phosphate-chitosan-biodegradable fiber scaffolds. Biomaterials 31: 840–847.
9. Tsai SP, Hsieh CY, Hsieh CY, Wang D, Huang LL, et al. (2007) Preparation and Cell Compatibility Evaluation of Chitosan/Collagen Composite Scaffolds Using Amino Acids as Crosslinking Bridges. J Appl Polym Sci 105: 1774-1785.
